# Flexible Bioinspired Healable Antibacterial Electronics for Intelligent Human‐Machine Interaction Sensing

**DOI:** 10.1002/advs.202305672

**Published:** 2023-12-22

**Authors:** Kuo Liu, Mingcheng Wang, Chenlin Huang, Yue Yuan, Yao Ning, Liqun Zhang, Pengbo Wan

**Affiliations:** ^1^ College of Materials Science and Engineering, State Key Laboratory of Organic–Inorganic Composites Beijing University of Chemical Technology Beijing 100029 China

**Keywords:** healable antibacterial elastomer, intelligent human‐machine interface, MXene, skin bionic flexible electronics, wearable ultrasensitive diagnostic healthcare sensing

## Abstract

Flexible electronic sensors are receiving numerous research interests for their potential in electronic skins (e‐skins), wearable human‐machine interfacing, and smart diagnostic healthcare sensing. However, the preparation of multifunctional flexible electronics with high sensitivity, broad sensing range, fast response, efficient healability, and reliable antibacterial capability is still a substantial challenge. Herein, bioinspired by the highly sensitive human skin microstructure (protective epidermis/spinous sensing structure/nerve conduction network), a skin bionic multifunctional electronics is prepared by face‐to‐face assembly of a newly prepared healable, recyclable, and antibacterial polyurethane elastomer matrix with conductive MXene nanosheets‐coated microdome array after ingenious templating method as protective epidermis layer/sensing layer, and an interdigitated electrode as signal transmission layer. The polyurethane elastomer matrix functionalized with triple dynamic bonds (reversible hydrogen bonds, oxime carbamate bonds, and copper (II) ion coordination bonds) is newly prepared, demonstrating excellent healability with highly healing efficiency, robust recyclability, and reliable antibacterial capability, as well as good biocompatibility. Benefiting from the superior mechanical performance of the polyurethane elastomer matrix and the unique skin bionic microstructure of the sensor, the as‐assembled flexible electronics exhibit admirable sensing performances featuring ultrahigh sensitivity (up to 1573.05 kPa^−1^), broad sensing range (up to 325 kPa), good reproducibility, the fast response time (≈4 ms), and low detection limit (≈0.98 Pa) in diagnostic human healthcare monitoring, excellent healability, and reliable antibacterial performance.

## Graphical abstract

A skin bionic healable antibacterial electronics is fabricated by assembling a newly prepared healable, recyclable, and antibacterial polyurethane elastomer with MXene nanosheets‐coated microdome array microstructures and an interdigitated electrode face‐to‐face, exhibiting ultrahigh sensitivity (up to 1573.05 kPa^−1^), broad sensing range (up to 325 kPa), low detection limit (≈0.98 Pa), excellent healability and antibacterial performance for sensitively intelligent human‐machine interaction sensing.

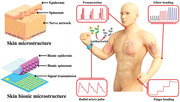

## 1 Introduction

Flexible electronic sensors have aroused extensive attention over past decades for their portability, flexibility, real‐time sensing response, and low detection limit in multifunctional e‐skins, intelligent robotics, wearable human‐machine interfacing, and healthcare monitoring.^[^ ^1^, ^2^, ^3^, ^4^, ^5^, ^6^ ^]^ Although great advancement has been achieved in flexible electronic sensors, there are still tremendous difficulties for flexible electronic sensors in simultaneously achieving ultrahigh sensing sensitivity, wide working range, and fast response/recovery.^[^ ^7^, ^8^, ^9^ ^]^ The reported flexible electronic sensors are mostly obtained from the conductive filler‐incorporated sensing materials layer between two electrodes, exhibiting relatively low sensing sensitivity, and narrow sensing range, due to the limited contact resistance variation between the sensing materials and their contact electrodes, the relatively limited resistance change of the sensing materials, the poor mechanical properties and material modulus of the matrix material to deform effectively under the external broad pressure range, and the compressed contact saturation or damage of the flexible electronic sensor under external large pressure loading.^[^ ^10^, ^11^ ^]^ Human skin, featured with the epidermis as the protection layer, the spinous microstructure as the sensing layer, and the nerve conduction network as the signal transmission layer, is capable of highly sensitive detecting subtle pressure (down to ≈ 1 Pa) by amplifying the external tactile stimuli from the spinous microstructure and transferring it to the underlying nearby mechanoreceptors and nerve conduction network, as well as achieving tactile perception of external stimuli over a wide range (up to ≈100–300 kPa).^[^ ^12^, ^13^, ^14^ ^]^ Recently, the emergence of biological skin bionics has provided great inspiration for the realization of novel flexible electronics with advanced multifunctionality and sensitively tactile perception functions.^[^ ^15^, ^16^, ^17^, ^18^ ^]^ Inspired by the human skin microstructure with excellent sensing performance, it is vitally important to develop a skin‐bionic flexible electronic sensor with a skin‐like microstructure to significantly improve the contact resistance change between the spinous microstructures‐contained sensing layer and the signal transmission layer under a broad pressure range to achieve ultrahigh sensing sensitivity, broad sensing range, and fast response. A novel 2D conductive nanomaterial, MXene possesses excellent mechanical strength, high electrical conductivity, large specific surface area, and rich surface hydrophilic groups (e.g., −OH,═O, −F),^[^ ^19^, ^20^, ^21^ ^]^ which is widely used in sensors,^[^ ^22^, ^23^ ^]^ batteries,^[^ ^24^ ^]^ supercapacitors,^[^ ^25^ ^]^ optoelectronics,^[^ ^26^ ^]^ biomedicine,^[^ ^27^ ^]^ electromagnetic interference (EMI) shielding,^[^ ^28^ ^]^ and water purification.^[^ ^29^ ^]^ The conductive nanomaterial MXene can be dip‐coated, spin‐coated, drop‐cast, or sprayed onto the elastomeric polymer substrates as the conductive sensing layer for flexible electronic sensors. Therefore, it is great necessary to design and assemble flexible wearable electronic sensors with a skin‐like microstructure to achieve high sensitivity, broad sensing range, and fast response perception of external pressure for ultrasensitively realizing the real‐time monitoring of the full‐range physiological signals of the human body.

The functional polymer materials, such as polyurethane, polydimethylsiloxane, polyimide, and Ecoflex, were commonly employed as polymer matrix to prepare flexible electronic sensors,^[^ ^30^, ^31^, ^32^ ^]^ which mostly have permanent crosslinking networks that cannot be repaired after sustaining mechanical damage (e.g., tension, collision, and scratch, etc.), reprocessed and recycled.^[^ ^33^, ^34^ ^]^ Therefore, the design of dynamic covalent bonds (e.g., boroxines, disulfide bonds, Diels‐Alder (DA) adducts, etc.) or noncovalent bonds (e.g., ionic interactions, hydrogen bonds, *π–π* interactions, metal‐ligand coordination, van‐der Waals interactions, host‐guest interactions, etc.) in polymer chains to endow polymer materials with healing and recycling capabilities, has attracted numerous research attention.^[^ ^35^, ^36^, ^37^, ^38^, ^39^ ^]^ Healable capability can efficiently repair unexpected mechanical damage of materials, restore the critical functions, and effectively extend the service life of materials.^[^ ^37^, ^38^ ^]^ Flexible electronic sensors based on a healable elastomeric polymer matrix, can achieve reliable and repeatable healable function, making it possible to restore the mechanical and electrical properties of damaged sensors.^[^ ^40^, ^41^ ^]^ However, the currently reported healable elastomers generally possess low healing efficiency and long healing time, which limits their potential application in flexible electronic sensors.^[^ ^42^, ^43^, ^44^ ^]^ Therefore, it is still a major challenge to prepare an elastomer matrix with superior mechanical strength and reliable healing efficiency simultaneously for flexible electronic sensors. In addition, because of the discomfort for the human skin (e.g., inflammation and itching) by the generated enormous bacteria from the long‐term contact between human skin and flexible wearable sensors,^[^ ^45^, ^46^ ^]^ the development of polymer matrix for flexible wearables with outstanding biocompatibility and reliable antibacterial properties is vitally desired. Thus, it is greatly urgent to develop multifunctional flexible electronic sensors with ultrahigh sensitivity, broad sensing range, fast response, efficient healing capability, and reliable antibacterial functions, which can realize accurate monitoring of full‐range human healthcare, effectively improve the service life of the flexible electronic sensors, and promote the practical applications in intelligent e‐skins, smart diagnostic healthcare sensing, intelligent theranostics, and wearable human‐machine interfacing.

**Scheme 1:**
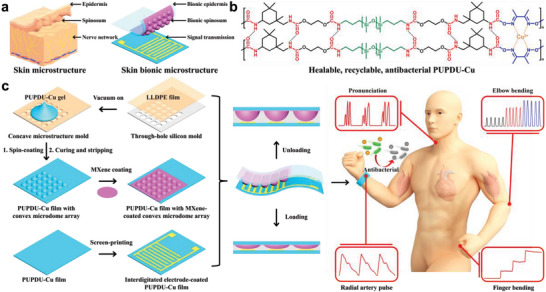
Schematic illustration of the preparation of a skin bionic ultrasensitive multifunctional flexible electronic sensor with excellent healability and reliable antibacterial function. a) Schematic of a skin bionic multifunctional flexible electronic sensor with a human skin‐like microstructure. b) Molecular structure of the newly prepared healable, recyclable, and antibacterial polyurethane elastomer (PUPDU‐Cu). c) Fabrication of the skin bionic ultrasensitive multifunctional flexible electronic sensor with reliable antibacterial capability for personalized healthcare monitoring and intelligent human‐machine interface.

Herein, bioinspired by the greatly sensitive human skin microstructure with the epidermis as the protection layer, the spinous microstructure as the sensing layer, and the nerve conduction network as the signal transmission layer, we successfully prepare a wearable skin bionic multifunctional flexible electronic sensor (**Scheme** **1**), in which a newly prepared healable, recyclable, and antibacterial polyurethane elastomer matrix with conductive MXene nanosheets‐coated microdome array serves as the bionic protective epidermis layer/the bionic spinous microstructure‐contained sensing layer, and a conductive interdigitated electrode‐coated polyurethane elastomer substrate serves as the signal transmission layer. The healable, recyclable, and antibacterial polyurethane elastomer matrix of the flexible electronic sensor functionalized with polysiloxane, Cu (II) ions‐coordinated dimethylglyoxime (DMG) and urethane groups (PUPDU‐Cu), is newly prepared by polycondensation reaction from bis(3‐aminopropyl)‐terminated poly(dimethylsiloxane) (NH_2_‐PDMS‐NH_2_, Mn = ≈3000), ethylene carbonate (EC), isophorone diisocyanate (IPDI) and DMG with the addition of copper (II) ions catalyzed by dibutyltin dilaurate. The healability of the polyurethane elastomer PUPDU‐Cu mainly depends on triple dynamic bonds, which are reversible hydrogen bonds, oxime carbamate bonds, and copper (II) ion coordination bonds with the nitrogen atoms of adjacent oxime groups in DMG. The as‐synthesized PUPDU‐Cu elastomer possesses a robust crosslinking network, superior mechanical strength at room temperature, and reliable healing efficiency (up to 94.5% for 6 h at 60 °C). The Cu^2+^ ions also have broad‐spectrum antibacterial activity, providing remarkable antibacterial properties for PUPDU‐Cu elastomer against *E. coli* and *S. aureus* at 95.5% and 92.0% respectively, to efficiently suppress the bacteria reproduction in contact position between the flexible electronic sensor and human skin. The assembled sensor exhibits greatly improved sensing performance with ultrahigh sensitivity (up to 1573.05 kPa^−1^), broad sensing range (up to 325 kPa), good reproducibility, low detection limit (≈0.98 Pa), and fast response (≈4 ms) for sensitively realizing the full‐range human healthcare monitoring with excellent healability and reliable antibacterial performance, demonstrating promising potential in next‐generation e‐skins, personalized healthcare detection, smart disease diagnosis, and wearable human‐machine interfacing.

## 2 Results and Discussion

The healable antibacterial PUPDU‐Cu elastomer (Scheme 1b), was facilely synthesized by polycondensation reaction from bis(3‐aminopropyl)‐terminated poly(dimethylsiloxane) (NH_2_‐PDMS‐NH_2_, Mn = ≈3000) (BAP‐PDMS), ethylene carbonate (EC), isophorone diisocyanate (IPDI) and dimethylglyoxime (DMG) with the addition of copper (II) ions catalyzed by dibutyltin dilaurate (Figure S1, Supporting Information). NH_2_‐PDMS‐NH_2_ was chosen as the soft segment to provide favorable flexibility and facilitate chain movement, thereby promoting better repair. IPDI was chosen as the hard segment ascribing to its bulky structure to inhibit the crystallization of the PUPDU‐Cu polymer networks and the steric influence of the cyclohexyl ring to further improve the mobility of the PUPDU‐Cu polymer networks. More importantly, the reversible oxime carbamate bonds could be provided from the chain extender DMG to facilitate healing performance. Furthermore, the addition of Cu^2+^ ions could form chelates with the nitrogen atoms of the adjacent oxime groups in DMG^[^ ^39^ ^]^ and the Cu^2+^ ions of the PUPDU‐Cu elastomer were able to disrupt the bacterial cell membrane, interact with the intracellular proteins and nucleic acids functional groups, and induce DNA damage to cause bacterial cell death and provide antibacterial activity.^[^ ^47^, ^48^ ^]^ Finally, the mainly existing triple dynamic bonds in PUPDU‐Cu elastomer, including reversible oxime carbamate bonds, hydrogen bonds, and copper (II) ion coordination bonds, could efficiently facilitate the excellent healing capability of the PUPDU‐Cu elastomer.

As shown in Figure S2 (Supporting Information) for ^1^H NMR spectrum and Figure S3 (Supporting Information) for ^13^C NMR of the PUPDU‐Cu elastomer, the characteristic peaks of BAP‐PDMS, EC, IPDI, and DMG could be observed, which confirmed the successful preparation of PUPDU‐Cu elastomer.^[^ ^43^ ^]^ As shown in Figure S4 (Supporting Information) for the Fourier transform infrared spectroscopy (FTIR), the peaks at 3324 cm^−1^ (N─H stretching vibration) and 1704  cm^−1^ (C═O stretching vibration) could be observed, demonstrating the generation of urethane groups in the PUPDU‐Cu elastomer. The negligible peaks at 2264 cm^−1^ (N═C═O stretching vibration) indicated that IPDI had been fully reacted (Figure S4, Supporting Information).^[^ ^49^, ^50^ ^]^ Figure S5 (Supporting Information) shows that the glass transition temperature (*T*_g_) of the PUPDU‐Cu elastomer was −124.48 °C. The lower glass transition temperature facilitated the movement of the molecular chains for promoting the healing of the PUPDU‐Cu elastomer in combination with the triple dynamic bonds. As shown in Figure S6 (Supporting Information) for the thermogravimetric analysis (TGA), there was no obvious weight loss of the PUPDU‐Cu elastomer < 200 °C. There are three obvious weight loss stages of PUPDU‐Cu elastomer at 200–338, 338—425, and 425—717 °C, corresponding to the sublimation of the DMG produced by thermal decomposition of the oxime carbamate bonds of the chain, the hard segment and the soft segment, respectively, indicating that the PUPDU‐Cu elastomer had high thermal stability. As shown in Figure S7 (Supporting Information) for X‐ray photoelectron spectroscopy (XPS), the Cu 2p peak could not be observed for PUPDU (Figure S7a, Supporting Information), while the Cu 2p peak could be observed at 933.3 eV for PUPDU‐Cu elastomer, demonstrating the existence of the copper coordination complex peak and the presence of copper (II) ion coordination bonds. The peaks at 940.6 and 962.8 eV were observed respectively, indicating the existence of partly coordinated copper ions (Figure S7b, Supporting Information).^[^ ^39^ ^]^ The above XPS spectra confirmed that PUPDU‐Cu elastomer with copper (II) ion coordination bonds were successfully synthesized.

Scheme 1c shows the preparation of the skin bionic ultrasensitive multifunctional flexible electronic sensor with reliable antibacterial capability for personal healthcare monitoring and human‐machine interfacing. First, the linear low‐density polyethylene (LLDPE) film was adhered onto a through‐hole silicon mold on the tray of the spin‐coating machine under vacuum to form the concave microstructures as the template for further replication. Then the PUPDU‐Cu gel dissolved in tetrahydrofuran (THF) was dropped onto the surface of the LLDPE film with the concave microstructures under the spin‐coating with vacuum. The rapid volatilization of highly volatile THF during spin coating led to the curing of PUPDU‐Cu gel. After curing, the PUPDU‐Cu elastomer film with the convex microdome array microstructure was obtained after stripping it from the LLDPE film. Next, conductive MXene nanosheets were coated onto the surface of the PUPDU‐Cu elastomer film with the convex microdome array microstructure, which works as the protective epidermis layer/the sensing layer of the skin bionic sensor. The signal transmission layer of the skin bionic sensor was obtained by screen‐printing the conductive silver paste‐based interdigitated electrode onto the surface of the flat PUPDU‐Cu elastomer film. The skin bionic multifunctional flexible electronic sensor was facilely assembled from the MXene‐coated convex microdome array microstructure‐contained PUPDU‐Cu elastomer film and the interdigitated electrode‐coated PUPDU‐Cu elastomer film face‐to‐face.

**Figure 1:**
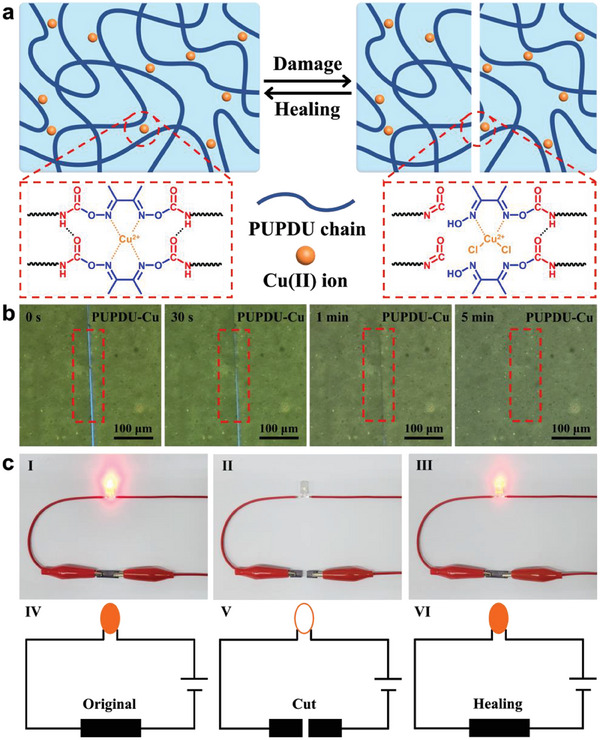
Healability of the PUPDU‐Cu elastomer. a) Schematic illustration for the healing of the PUPDU‐Cu elastomer. b) Optical microscope images of the healing of the scratch of the PUPDU‐Cu elastomer film. c) The circuit composed of the PUPDU‐Cu elastomer with the coated MXene conductive layer on the surface and orange LED bulb in series: (I) The original PUPDU‐Cu elastomer with the coated MXene conductive layer, (II) the completely cut PUPDU‐Cu elastomer with the coated MXene conductive layer, (III) the PUPDU‐Cu elastomer after healing, and (IV‐VI) the corresponding schematic circuits.

The healability of the PUPDU‐Cu elastomer was mainly determined by the reversible oxime carbamate bonds, hydrogen bonds, and copper (II) ion coordination bonds (**Figure** **1**a). As shown in Figure 1b, the PUPDU‐Cu elastomer film was cut out with a wideness at 7–12 µm by a blade, which could be observed under a microscope with a built‐in heating function. Along with the time evolution at 60 °C, the cut on the surface of the PUPDU‐Cu elastomer film almost disappeared and was gradually healed after 5 min. Figure S8 (Supporting Information) illustrates the optical microscope images of the healing of the cut of the PUPDU elastomer film. Figure 1c shows the circuit composed of the PUPDU‐Cu elastomer with the coated MXene conductive layer on the surface and orange LED bulb in series. the LED bulb was successfully lit under the power supply of 4.5 V battery. After cutting the PUPDU‐Cu elastomer film with the coated MXene conductive layer on the surface, the LED bulb was extinguished immediately. After the cut PUPDU‐Cu elastomer film with the coated MXene conductive layer on the surface was re‐contacted and heated at 60 °C, the damaged conducting layer would re‐connect to restore the conducting performance, and the LED bulb was successfully illuminated again.

Figure S9 (Supporting Information) shows the typical tensile stress‐strain curves of the PUPDU elastomer and the PUPDU‐Cu elastomer. It could be seen that the tensile strength of the PUPDU‐Cu elastomer (3.11 MPa) was much higher than that of the PUPDU elastomer (1.74 MPa). The tensile strain of the PUPDU‐Cu elastomer (386%) was more than twice of the PUPDU elastomer (164.6%), which was probably ascribed to the increased hidden length of more folded polymer chains from Cu^2+^ coordination bonds. Figure S10 (Supporting Information) displays the typical compressive stress‐strain curves of the PUPDU‐Cu elastomer. It could be seen that the PUPDU‐Cu elastomer could withstand multiple compression‐relaxation cycles at a compression strain of 50% with an insignificant decrease in compressive stress, demonstrating that the PUPDU‐Cu elastomer had repeatable compressibility and excellent elasticity. Thus, the excellent tensile and compressive properties of PUPDU‐Cu elastomer further confirmed their great potential in high‐performance flexible electronic sensors. Figures S11a,b (Supporting Information) show the tensile stress‐strain curves of the dumbbell‐shaped PUPDU elastomer spline and the dumbbell‐shaped PUPDU‐Cu elastomer before and after cutting, and after contacting and healing at 60 °C for different time. It could be observed that along with the time evolution, the mechanical properties of the PUPDU elastomer and the PUPDU‐Cu elastomer gradually recovered (Figure S11a–c, Supporting Information), and the healing efficiency of the PUPDU‐Cu elastomer was up to ≈56.7% and ≈94.5% at 60 °C for 15 min and 6 h, respectively. (Figure S11d, Supporting Information). The mechanical performances recovered rapidly with the recombination of dynamic covalent bonds and noncovalent bonds in the elastomers. The PUPDU‐Cu elastomer could be recycled many times and still maintain the mechanical robustness. As shown in Figure S12 (Supporting Information), the PUPDU‐Cu elastomer film was broken into pieces and homogeneously dissolved in THF. Afterward, the obtained solution was cast in a polytetrafluoroethylene (PTFE) mold, and the defect‐free PUPDU‐Cu elastomer film was obtained after evaporation of the THF solvent. Compared with the pristine PUPDU‐Cu elastomer film, the PUPDU‐Cu elastomer film after three times of recycling could still maintain the mechanical robustness, indicating that the PUPDU‐Cu elastomer had good recycling and reprocessing capabilities (Figure S13, Supporting Information).

**Figure 2:**
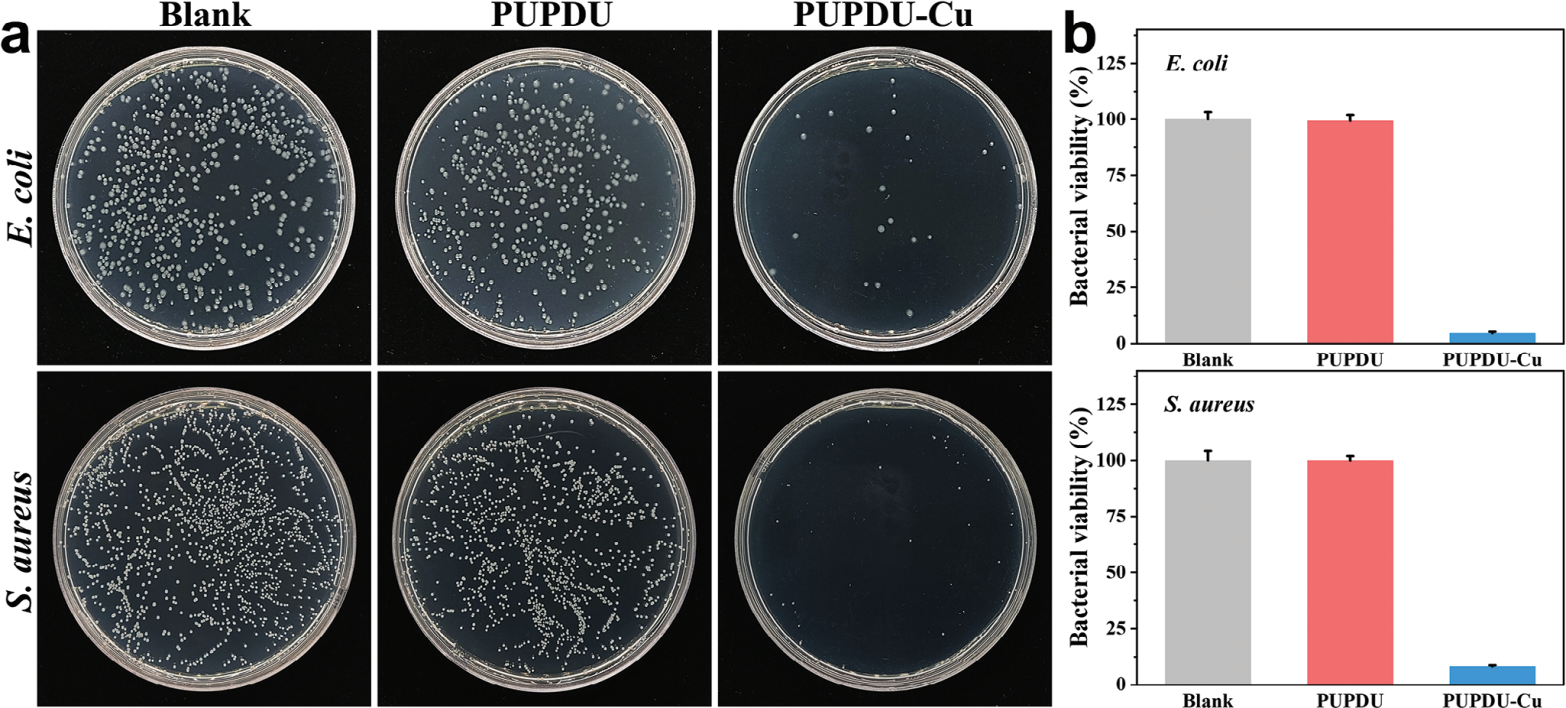
a) Photographs of the survival bacterial colonies on agar plates to demonstrate in vitro antibacterial activities of the PUPDU and PUPDU‐Cu elastomers against *E. coli* and *S. aureus* respectively. b) The bacterial viability of the PUPDU and PUPDU‐Cu elastomers against *E. coli* and *S. aureus* respectively.

Because of the direct contact of the PUPDU‐Cu elastomer matrix with human skin in flexible electronic sensors, the biocompatibility of the PUPDU‐Cu elastomer is vitally important. The cytotoxicity of PUPDU‐Cu elastomer was evaluated by culturing the PUPDU‐Cu elastomer extract with L929 cells for a certain time by the Cell Counting Kit‐8 (CCK‐8) test. Figure S14 (Supporting Information) shows the cell proliferation of L929 cells after being cultured for 24, 48, and 72 h respectively. In comparison to the blank control groups, the number of L929 cells cultured with PUPDU‐Cu elastomer extract did not change significantly and was in a healthy fusiform morphology, indicating that the PUPDU‐Cu elastomer had no obvious cytotoxicity. In addition, with the evolution of the culture time, the cell viability of L929 cells cultured with the PUPDU‐Cu elastomer extract remained at ≈ 90% after 72 h (Figure S15, Supporting Information), further demonstrating the good biocompatibility of the PUPDU‐Cu elastomer. In addition, Cu^2+^ ions possess broad‐spectrum bactericidal properties against bacteria, fungi, and viruses, showing great potential in medical applications. We evaluated the antibacterial activity of the PUPDU‐Cu elastomer against Gram‐negative *E. coli* and Gram‐positive *S. aureus*. After the PUPDU‐Cu elastomer was co‐cultured with the bacterial suspension for a certain time, it could be observed that the number of the surviving bacteria of both Gram‐negative *E. coli* and Gram‐positive *S. aureus* was remarkably less than the blank control group (**Figure** **2**a). At the same time, the quantitative analysis demonstrates that the bactericidal efficacy of PUPDU‐Cu elastomers against Gram‐negative *E. coli* and Gram‐positive *S. aureus* were 95.5% and 92.0%, respectively, while the PUPDU elastomer without Cu^2+^ ions had almost no antibacterial activity (Figure 2b). Thus, the PUPDU‐Cu elastomer had reliable antibacterial performance, which is mainly attributed to that the Cu^2+^ ions of the PUPDU‐Cu elastomer could lead to bacterial death by disrupting bacterial cell membrane, interacting with the intracellular proteins and nucleic acids functional groups, causing protein aggregation and inducing DNA damage in a series of processes to result in the loss of the ability of bacteria to divide and proliferate.^[^ ^47^, ^48^ ^]^

**Figure 3:**
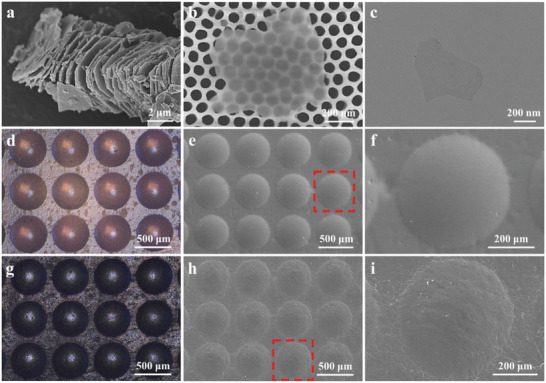
The morphology of MXene nanosheets and the microdome array microstructures. a) SEM image of the accordion‐like Ti_3_C_2_T_x_, derived from Ti_3_AlC_2_ by selective etching of Al layers in LiF‐HCl etchant. b) SEM image of the MXene nanosheet. c) Transmission electron microscopy (TEM) image of the MXene nanosheet. d) Optical image of the microdome array microstructures without MXene coating. e) SEM image of the microdome array microstructures without MXene coating (scale bar: 500 µm). f) SEM image of the microdome array microstructures without MXene coating (scale bar: 200 µm). g) Optical image of the conductive MXene nanosheets‐coated microdome array microstructures. h) SEM image of the conductive MXene nanosheets‐coated microdome array microstructures (scale bar: 500 µm). i) SEM image of the conductive MXene nanosheets‐coated microdome array microstructures (scale bar: 200 µm).

**Figure** **3**a displays the scanning electron microscopy (SEM) image of the accordion‐like Ti_3_C_2_T_x_ obtained after selectively removing the monoatomic Al layers of the MAX phase (Ti_3_AlC_2_) (Figure S16, Supporting Information).^[^ ^51^ ^]^ The MXene nanosheets were obtained after ultrasonic treatment of Ti_3_C_2_T_x_ (Figure 3b,c). The thickness of MXene nanosheet was ≈ 1.7 nm from Figure S17b (Supporting Information) for the atomic force microscopy (AFM) image. The successful removal of monoatomic Al layers was proved by the left‐shifted characteristic (002) peak and the weakened characteristic (104) peak in X‐ray diffraction (XRD) patterns (Figure S18, Supporting Information). As depicted in Figure S19 (Supporting Information) for the X‐ray photoelectron spectroscopy (XPS) spectrum, MXene nanosheets mainly contained C, F, Ti, and O elements, displaying the formation of the rich surface functional groups (e.g., ─OH,═O, ─F, etc.) in MXene nanosheets to endow MXene nanosheets with excellent hydrophilicity, which consequently could be well combined with the PUPDU‐Cu polymer substrate through supramolecular interactions (Figure S20, Supporting Information).

The convex microdome array microstructure‐contained PUPDU‐Cu elastomer film was successfully prepared by using the through‐hole silicon mold and the dimension parameters of the through‐hole silicon mold are shown in Figure S21 (Supporting Information). After attaching LLDPE film onto the through‐hole silicon mold on the spin‐coating machine under vacuum, the concave microstructures were formed as the template for further replication. After dropping the PUPDU‐Cu gel dissolved in THF onto the above as‐obtained concave microstructures‐contained LLDPE film with the spin‐coating under vacuum for curing, the convex microdome array microstructure‐contained PUPDU‐Cu elastomer film was obtained after peeling it from the LLDPE film. Figure 3d shows the optical microscope image of the obtained microdome array microstructures without MXene coating. Figure 3e,f present the SEM images of the obtained microdome array microstructures without MXene coating. The convex microdome array microstructure‐contained PUPDU‐Cu elastomer film was coated with the conductive MXene nanosheets and worked as the protective epidermis layer/the sensing layer of the skin bionic sensor. Figure 3g illustrates the optical microscope image of the conductive MXene nanosheets‐coated microdome array microstructures. Figures 3h,i present the SEM images of the conductive MXene nanosheets‐coated microdome array microstructures. The coated MXene nanosheet layers on the microdome array microstructures could form the conducting sensing layers for further healthcare sensing. The cross‐section of the conductive MXene nanosheets‐coated microdome array structure is shown in Figure S22 (Supporting Information). The height of the microdome was ≈ 100 µm, and the distance between the adjacent two microdomes was ≈ 70 µm. The detailed size of the conductive interdigitated electrode is shown in Figure S23a (Supporting Information). Figure S23b,c (Supporting Information) were the optical photograph and the SEM images of the interdigitated electrode‐coated PUPDU‐Cu elastomer film.

**Figure 4:**
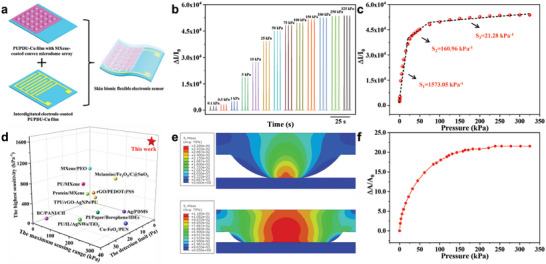
The sensing performance of the flexible electronic sensor. a) The schematic of the assembly of the sensor. b) The sensing responses of the sensor under different external pressures. c) The sensing performances of the sensor under external pressures up to 325 kPa. d) The comparison of the sensing performance for the skin bionic flexible electronic sensor with that for the previous works reported in literatures^[^ ^2^, ^3^, ^4^, ^6^, ^7^, ^8^, ^10^, ^11^, ^12^, ^13^, ^14^ ^]^ (Table S1, Supporting Information). e) The finite‐element simulation of stress distribution of microdome structure under the pressures of 32 and 118 kPa respectively. f) The relative area change (Δ*A*/*A*_0_) between the microdome array structure and the flat electrode under different pressures calculated by finite‐element simulation.

**Figure** **4**a displays that the flexible electronic sensor was assembled face‐to‐face from a PUPDU‐Cu elastomer film with the conductive MXene nanosheets‐coated microdome array microstructures and an interdigitated electrode‐coated PUPDU‐Cu elastomer film. Figure S24 (Supporting Information) shows the photographs of bending and twisting the as‐assembled flexible electronic sensors with reliable flexibility. Figures S25 and S26 (Supporting Information) show the sensing performances and the simulated results of the flexible electronic sensors prepared from the different hole diameters of the through‐hole silicon molds. It could be seen that the microdome array microstructures for the flexible electronic sensor prepared from the hole diameter of the through‐hole silicon mold at 500 µm had the best sensing performance. For the sensors with the microdome array microstructures prepared from the smaller hole diameters of the through‐hole silicon molds, the compressive deformation saturation under external pressure was rapidly achieved (Figure S25, Supporting Information), resulting in relatively smaller contact area change, lower sensing response, and sensing performance. For the sensor with the microdome array microstructures prepared from the larger hole diameters of the through‐hole silicon molds, a relatively higher initial contact area was observed (Figure S25, Supporting Information), resulting in lower sensing response and sensing performance. Furthermore, different amounts of MXene nanosheets were coated onto the surface of the microdome array microstructures‐contained PUPDU‐Cu elastomer film prepared from the through‐hole silicon mold with 500 µm hole diameter for the flexible electronic sensor, and the optimal sensing response could be observed with 0.24 mg MXene nanosheets coating (Figure S27, Supporting Information), because less MXene nanosheets coating cannot establish effective conductive pathway and network, while more MXene nanosheets coating could cover up the microdome array microstructure, leading to limited contact area change and lower sensing response. The sensing responses of the flexible electronic sensor under varied external pressures can be observed in Figure 4b.

Figure 4c shows the sensing performances of the flexible electronic sensor under external pressures, exhibiting a wide sensing range up to 325 kPa. The sensing sensitivity (S = δ(Δ*I*/*I*_0_)/δP) could be observed in Figure 4c, demonstrating the ultrahigh sensing sensitivity up to 1573.05 kPa^−1^. Figure S28 (Supporting Information) shows the sensing performances of the flexible electronic sensors assembled with different sensing layers and different electrodes, suggesting that the highest sensing performance was able to be gained from the flexible electronic sensor assembled with MXene nanosheets‐coated microdome array microstructures‐contained PUPDU‐Cu elastomer film and the interdigitated electrode‐coated PUPDU‐Cu elastomer film. The flexible electronic sensor (Figure S28a, Supporting Information) assembled from the MXene nanosheets‐coated flat PUPDU‐Cu elastomer film and the interdigitated electrode‐coated PUPDU‐Cu elastomer film, had relatively no obvious change in contact area between the MXene nanosheets‐coated flat PUPDU‐Cu elastomer film and the interdigitated electrode‐coated PUPDU‐Cu elastomer film under different external pressures, resulting in a relatively lower sensing response. The flexible electronic sensor (Figure S28b, Supporting Information) assembled from the PUPDU‐Cu elastomer film with the conductive MXene nanosheets‐coated microdome array microstructures and the two conductive stripes‐contained electrodes, had relatively smaller effective contact area change at the contact interface between the PUPDU‐Cu elastomer film with the conductive MXene nanosheets‐coated microdome array microstructures and the two conductive stripes‐contained electrodes under different external pressures, resulting in a relatively lower sensing sensitivity. For the flexible electronic sensor (Figure S28c, Supporting Information) assembled from the PUPDU‐Cu elastomer film with the conductive MXene nanosheets‐coated microdome array microstructures and the conductive planar electrode, the initially relatively large contact area at the contact interface between the PUPDU‐Cu elastomer film with the conductive MXene nanosheets‐coated microdome array microstructures and the conductive planar electrode, and the relatively limited contact area change under different external pressures, could be obtained, resulting in a relatively lower sensing sensitivity. For the flexible electronic sensor (Figure S28d, Supporting Information) assembled from two PUPDU‐Cu elastomer films with the conductive MXene nanosheets‐coated microdome array microstructures, the relatively larger initial contact area at the contact interface between two PUPDU‐Cu elastomer films with the conductive MXene nanosheets‐coated microdome array microstructures, resulting in a relatively lower sensing sensitivity. Compared to the above sensors, the flexible electronic sensor (Figure 4a,c; Figure S28e,f, Supporting Information) assembled from the PUPDU‐Cu elastomer film with the conductive MXene nanosheets‐coated microdome array microstructures and the interdigitated electrode‐coated PUPDU‐Cu elastomer film, exhibited the highest sensing sensitivity due to the extremely small initial contact area and the relatively large effective contact area change at the contact interface between the PUPDU‐Cu elastomer film with the conductive MXene nanosheets‐coated microdome array microstructures and the interdigitated electrode. Figure S29 (Supporting Information) shows the tensile stress‐strain curves of the TPU/5%BN elastomer. Figure S30 (Supporting Information) demonstrates the sensing performances of the skin bionic flexible sensors prepared from different elastomer matrixes with variable elastic moduli (Ecoflex (≈0.044 MPa), PUPDU (≈2.24 MPa), TPU (≈3.90 MPa), and TPU/5 wt.% Boron Nitride (TPU/5%BN) (≈4.60 MPa)), respectively. It could be seen that the flexible electronic sensor prepared from PDPDU‐Cu elastomer matrix (elastic modulus at ≈3.15 MPa), exhibited relatively higher sensing sensitivity and broader sensing range (Figure 4c; Figure S31, Supporting Information) simultaneously. This may be related to the compression deformation degree and the relative contact area change of the sensors prepared from different elastomers with different moduli under external pressure. The compressive deformation saturation at the contact interface under the same external pressure, could be rapidly achieved from the sensors prepared from the elastomer matrixes with relatively lower elastic moduli than PDPDU‐Cu elastomer matrix, demonstrating higher relative contact area change, higher sensing sensitivity, and narrow sensing range. The relatively smaller compression deformation at the contact interface under same external pressure, could be obtained from the sensors prepared from the elastomer matrixes with relatively higher elastic moduli than PDPDU‐Cu elastomer matrix, resulting in a lower relative area change, relatively lower sensing sensitivity, and broader sensing range for bearing larger external pressure.

The sensor in Figure 4a had a real‐time output current sensing response, showing a good match with the input pressure (Figure S32, Supporting Information), extremely fast response/recovery time (4/7 ms) (Figure S33, Supporting Information), attributing to the lower viscoelasticity, fast deformation capability, excellent resilience, and low hysteresis of the elastomers and the faster efficient conducting sensing channel of the sensor. Figure S34 (Supporting Information) displays the sensing responses of the flexible electronic sensor under the cyclic pressure loading/unloading at different frequencies with a stable signal output at different compression frequencies, suggesting that the sensor can stably work under different pressure frequencies. The cycling sensing stability of the flexible electronic sensor is shown in Figure S35 (Supporting Information), demonstrating the reliable mechanical and sensing durability of the sensor for long‐term stable human motion detection. The *I*–*V* curves of the sensor at different external pressures demonstrated the perfect ohmic contact behavior between the conductive MXene nanosheets‐coated microdome array microstructures and the interdigitated electrode at various applied pressures (Figure S36, Supporting Information). Figure S37 (Supporting Information) shows the sensing performances of the original sensor and the sensor after scratching and after healing to finger bending respectively, indicating that the sensor had reliable healability from the healable PUPDU‐Cu elastomer for the reliable long‐term healthcare monitoring. As shown in Figure 4d, the obtained skin bionic flexible electronic sensor demonstrated significantly improved sensing performance compared to that for the flexible electronics reported in the literatures.^[^ ^2^, ^3^, ^4^, ^6^, ^7^, ^8^, ^10^, ^11^, ^12^, ^13^, ^14^ ^]^

The microdome array microstructure could provide a large specific surface area, resulting in the effective compression deformation between the sensing layer and the signal transmission layer under external pressure. At a fixed voltage, the increase in contact area resulted in the increase of current. After the external pressure was unloaded, both the sensing layer and the signal transmission layer returned to the original state, and the decrease in contact area led to the decrease of current. The equivalent circuit simulation model was established to further reveal the sensing mechanism (Figure S38, Supporting Information), demonstrating that the sensing response Δ*I*/*I*_0_ of the sensor to external pressure was mainly attributed to the contact resistance change between the PUPDU‐Cu elastomer film with the conductive MXene nanosheets‐coated microdome array microstructures and the interdigitated electrode‐coated PUPDU‐Cu elastomer film of the sensor caused by the corresponding contact area change under a fixed voltage, which could be expressed as: Δ*I*/*I*_0_ ≈ Δ*A*/*A*_0_ (Equations S(1)–S(3), Supporting Information).^[^ ^52^ ^]^

Figure 4e shows the finite‐element analysis (FEA) of the corresponding stress distribution of the microdome array microstructure under the pressures of 32 and 118 kPa, respectively. The contact area change (Δ*A*) between the microdome array microstructure and the contacted electrode under external pressure loading was extracted from the FEA result, and the relative contact area change (Δ*A*/*A*_0_) was further calculated (Figure 4f). The stress was distributed nonuniformly, and because of the relatively smaller contact area, the stress concentration was observed to be near the contacting tips (Figure 4e), leading to the relatively higher mechanical deformation and the fast relative contact area change. Along with the increasing externally loaded pressures, the mechanical deformation for the microdome array microstructure was observed to be gradually saturated, and the slow relative contact area change could be observed under further additional loaded pressure from compressing the microdome array microstructure with the relatively bigger cross‐sectional area. It can be found from S = δ(Δ*I*/*I*_0_)/δP ≈ δ(Δ*A*/*A*_0_)/δP (Equation S(4), Supporting Information),^[^ ^52^ ^]^ under the same external pressure P loading, the sensor sensitivity is proportional to the relative contact area change between the top conductive MXene nanosheets‐coated microdome array microstructure sensing film and the bottom interdigitated electrode‐coated PUPDU‐Cu elastomer film. As shown in Figure 4f, under the external loaded pressures < 25 kPa, the largest slope of the relative contact area change curve could be observed, due to the obviously larger mechanical deformation of the microdome array microstructure from the lower initial contact area with the substrate, and the flexible electronic sensor exhibits the highest sensing sensitivity (Figure 4c). Under the loaded pressures of 25–75 kPa, the slope of the relative contact area change curve slowly decreased from the relatively lower mechanical deformation of the microdome array microstructure, and the sensing sensitivity of the sensor decreased (Figure 4c). Under the loaded pressures of 75–325 kPa, the slope of the relative contact area change curve further decreased due to the gradually saturated mechanical deformation of the microdome array microstructure, and the sensing sensitivity of the sensor further decreased (Figure 4c).

**Figure 5:**
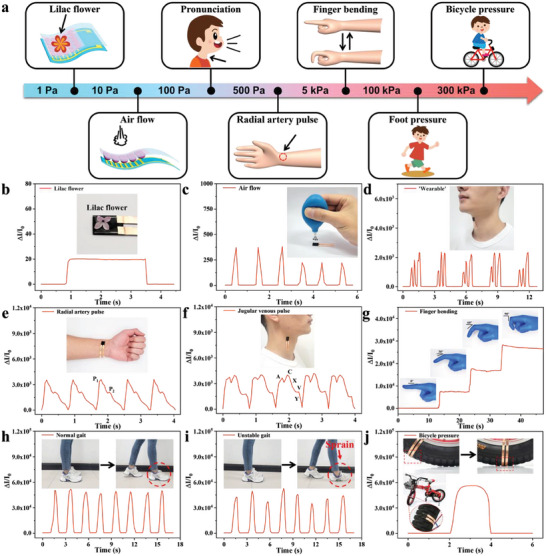
Versatile sensing applications of the flexible electronic sensor in various human motion detection. a) Schematic of the flexible electronic sensor in various human motion detection. b) Sensing performance of the flexible electronic sensor to a lilac flower. Inset: the optical image of a lilac flower (≈9.8 mg) placed on the sensor. c) Sensing performance of the flexible electronic sensor to airflow. Inset: the optical image of the airflow generated from the rubber suction bulb to the sensor. d) Sensing the performance of the flexible electronic sensor when speaking “wearable” from the volunteer. Inset: optical image of the sensor attached onto the throat of the volunteer. e) Sensing the performance of the flexible electronic sensor to the radial artery pulse. Inset: optical image of the sensor attached onto the wrist. f) Sensing the performance of the flexible electronic sensor to the jugular venous pulse. Inset: optical image of the sensor attached onto the neck. g) Sensing the performance of the flexible electronic sensor to different finger bending angles. Inset: optical images of the sensor attached onto a finger at different bending angles. h,i) Sensing performance of the flexible electronic sensor to h) normal gait and i) unstable gait. Inset: optical images of the normal and unstable gaits respectively. j) Sensing performance of the flexible electronic sensor to the loaded external pressure to the tire from the driving with the volunteer (a weight of 52 kg). Inset: the sensor is attached onto the bicycle tire for the loaded pressure sensing.

Benefiting from the high sensitivity, broad sensing range, and fast response/recovery, the flexible electronic sensor is helpful for real‐time sensitive human motion detection (**Figure** **5**a). Figure 5b shows the sensing performance of the flexible electronic sensor to a lilac flower (≈0.98 Pa), indicating that the sensor had a relatively low detection limit for sensitively detecting ultralow pressure. Figure 5c shows the real‐time sensing performance of the tiny airflow generated from the rubber suction bulb to the sensor. The pronunciation recognition could be gained by attaching the sensor onto the throat of a volunteer when speaking different words, such as “wearable” (Figure 5d) and “sensor” (Figure S39, Supporting Information), which could be distinguished by the different vibration generated from the throat pronunciation, showing great application potential in language expression for deaf‐mute people and information transmission. The radial artery pulse signal is a crucial indicator to assess heart rate and arterial conditions.^[^ ^53^, ^54^ ^]^ The flexible electronic sensor was tightly stuck onto a volunteer's wrist to real‐timely record the radial artery pulse, and the pulse signal frequency of the volunteer was calculated from Figure 5e to be 74 beats/min. The characteristic systolic peak (P_1_) and reflected peak (P_2_) can be observed in Figure 5e. As an important parameter for the diagnosis of arterial stiffness and vascular aging, the calculated radial enhancement index (P_2_/P_1_) was ≈0.59, which is consistent with the literature reference value for a healthy 25‐year‐old male (height of 180 cm).^[^ ^55^, ^56^ ^]^ Furthermore, the jugular vein pulse (JVP) is directly connected to the right atrium of the human heart, which can directly reflect the pressure and volume changes of the right atrium for providing more valuable medical information for the diagnosis of heart disease and heart failure.^[^ ^46^, ^57^ ^]^ As shown in Figure 5f, the jugular venous pulse frequency was calculated to be ≈75 beats min^−1^, which was in good agreement with the radial artery pulse frequency obtained in Figure 5e. At the same time, the characteristic JVP peaks of three positive peaks, including atrial contraction (A), tricuspid bulging (C), and atrial venous filling (V), and two negative peaks, including atrial relaxation (X) and ventricular filling (Y), could be observed in Figure 5f. The obtained human radial and jugular pulse signals are vitally promising in the medical diagnosis of various cardiovascular diseases, such as arterial stiffness and heart failure.^[^ ^57^, ^58^ ^]^

As displayed in Figure 5g, the flexible sensor was employed to real‐timely monitor finger bending at varied angles, showing great potential in the versatile detection of gesture expression and postoperative rehabilitation training. Figure S40 (Supporting Information) shows the sensing performance of the flexible electronic sensor to elbow bending at different angles. Interestingly, the human gait could be sensitively recognized by attaching the sensor onto the bottom of the forefoot of the volunteer. Figure 5h,i show the sensing performance of the flexible electronic sensor to normal gait and a simulated unstable gait respectively. Obviously, the sensing performance of the walking gait of a healthy volunteer was stable, while that of the volunteer with the simulated gait disorders (e.g., sprain, toddle, and fracture) was unstable. Gait detection is of great significance for infant toddler gait care, and the postoperative rehabilitation training of patients and elderly persons with gait disorders. Furthermore, the flexible sensor was employed for the loaded pressure detection by attaching the sensor onto the bottom of a bicycle tire. A volunteer with a weight of 52 kg was slowly driving the bicycle forward and the pressure loaded on the related bicycle tire could be monitored when the sensor attached onto the tire was in contact with the ground (Figure 5j), indicating that the flexible electronic sensor could facilely detect higher loaded pressures.

**Figure 6:**
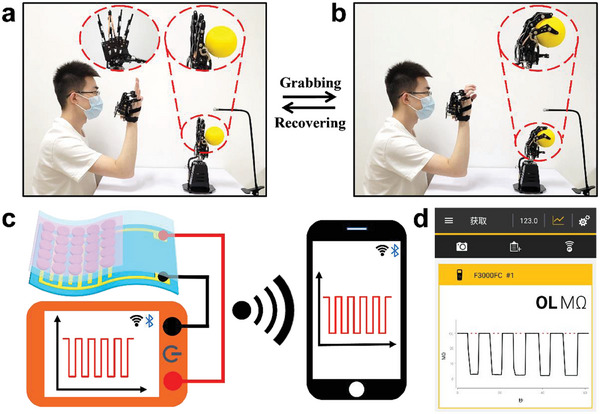
Wearable human‐machine interface and wireless pressure sensing. a,b) Photographs of the flexible electronic sensor attached onto the front part of the manipulator's ring finger for reversibly wireless pressure monitoring when reversibly grabbing the yellow ball by the manipulator remotely operated by a somatosensory mechanical glove. Inset: the enlarged images of the manipulator. c) The scheme of the wireless sensing performance transmission by combining the flexible electronic sensor with the wireless transmitter to send the sensing signal to a mobile phone. d) The obtained real‐time sensing performance for reversibly grasping the yellow ball.

Moreover, the emergence of advanced comfortable wearables provides a promising platform for the vigorous development of human‐machine interfaces. The human‐machine interaction system with integrated flexible electronics could facilitate to satisfy the requirements of users, which is an inevitable development trend in the future.^[^ ^59^, ^60^ ^]^ In order to achieve the effectiveness and the accuracy of information interaction in wearable human‐machine interfaces, it is crucial to develop multifunctional flexible electronics with excellent flexibility and sensing performance. The flexible electronic sensor was employed to wireless intelligent human‐machine interface by attaching it onto the ring finger of a manipulator in connection with a wireless transmitter. As shown in **Figure** **6**a,b, when the volunteer remotely controlled the manipulator by wearing a somatosensory mechanical glove to repeatedly grab the yellow ball in front of the manipulator, the corresponding loaded pressure generated from the contact between the manipulator and the yellow ball could be detected in time. Meanwhile, the sensing performance could be wirelessly transmitted to a mobile phone by the combined wireless transmitter (Figure 6c,d). Above results indicated the flexible electronic sensor had great application prospects in intelligent electronic skins and wearable human‐machine interfaces.

## 3 Conclusion

In conclusion, we proposed a skin‐bionic multifunctional flexible electronic sensor based on a newly prepared healable, recyclable, and antibacterial polyurethane elastomer matrix functionalized with polysiloxane, Cu (II) ions‐coordinated dimethylglyoxime, and urethane groups. The elastomer matrix PUPDU‐Cu exhibited robust mechanical properties, highly improved healing efficiency, recyclable performance, robust biocompatibility, and reliable antibacterial capability against *E. coli* and *S. aureus* at 95.5% and 92.0% respectively. The flexible electronic sensor was facilely assembled from a PUPDU‐Cu elastomer film with conductive MXene nanosheets‐coated microdome array microstructures and an interdigitated electrode‐coated PUPDU‐Cu elastomer film face‐to‐face, showing superior sensing performance with ultrahigh sensitivity (up to 1573.05 kPa^−1^), broad sensing range (up to 325 kPa), good reproducibility, low detection limit (≈0.98 Pa) and fast response time (≈4 ms). The flexible electronic sensor was successfully employed to real‐time detection of the full‐range human healthcare, including subtle human activities (such as pulse, pronunciation, etc.) and large‐scale human activities (such as finger bending, gait detection, etc.). In addition, the flexible electronic sensor could be integrated with a smart manipulator and connected with a wireless transmitter to achieve wireless intelligent human‐machine interfacing. Furthermore, the flexible electronic sensor based on the newly prepared PUPDU‐Cu polyurethane elastomer matrix, exhibited excellent healability and reliable antibacterial performance, demonstrating great potential in improving the lifetime of the material and the sensor device, and in facilitating the biocompatible contact with human skin for further antibacterial and intelligent medical treatment. On the basis of the above line of research work, the skin bionic multifunctional flexible electronic sensor based on a healable, recyclable, and antibacterial elastomer matrix has great prospects in next‐generation artificial e‐skins, smart diagnostic healthcare sensing, intelligent theranostics, and wearable human‐machine interfacing.

## 4 Experimental Section

### Materials

Bis(3‐aminopropyl)‐terminated poly(dimethylsiloxane) (NH_2_‐PDMS‐NH_2_, Mn = ≈3000), Ethylene carbonate (EC, 98%) and Isophorone diisocyanate (IPDI, 99%) were purchased from Macklin Biochemical Co., Ltd. N, N‐Dimethylformamide (DMF, 99.5%), Dimethylglyoxime (DMG, 98%) and Lithium fluoride (LiF, 99.9%) were purchased from Aladdin Biomedical Technology Co., Ltd. The catalyst dibutyltin dilaurate (DBTDL, 97.5%) was purchased from J&K Scientific Co., Ltd. Copper (II) chloride (CuCl_2_, anhydrous, 98%) was purchased from Alfa Aesar Chemicals Co., Ltd. Tetrahydrofuran (THF, 99.5%) and Hydrochloric acid (HCl, 36.0‐38.0 wt.%) were purchased from Beijing Chemical Works. Ti_3_AlC_2_ was obtained from 11 Technology Co., Ltd. Linear low‐density polyethylene film (LLDPE) was obtained from Alibaba group. The through‐hole silicon molds with different hole diameters were supplied by Chongqing Yintao Laser Equipment Co., Ltd.

### Synthesis of the PUPDU‐Cu Elastomer

The PUPDU‐Cu elastomer matrix was synthesized in three steps. First, the amine‐terminated H_2_N‐PDMS‐NH_2_ (9.45 g) and EC (0.55 g) were added into a 100 mL flask and stirred at 100 rpm for 4 h at 80 °C under an inert nitrogen atmosphere, and then the system was distilled at 115 °C, −0.095 MPa and 100 rpm for 1 h. Second, the mixture was cooled to 75 °C, and IPDI (1.2598 g) and DBTDL (0.02 g) were added sequentially, and stirred at 250 rpm under an inert nitrogen atmosphere. Third, increasing the temperature of the system to 90 °C, DMG (0.2925 g) dissolved in DMF (4 mL) was added into the system and stirred at 350 rpm for 30 min. CuCl_2_ (0.23 g) dissolved in DMF (1 mL) was further added into the system and stirred at 350 rpm for 30 min.

### Cytotoxicity Test of the PUPDU‐Cu Elastomer

The cytotoxicity of the PUPDU‐Cu elastomer was assessed by the Cell Counting Kit‐8 (CCK‐8) test. First, 0.1 g PUPDU‐Cu elastomer was sterilized and added to 10 mL Dulbecco's Modified Eagle Medium (DMEM), and the PUPDU‐Cu elastomer extraction solution was obtained by filtration after soaking in DMEM at 37 °C for 24 h. Then, the PUPDU‐Cu elastomer extraction solution (1000 µL/well) and L929 cells (6 × 10^4^ cells/well) were added into the 24‐well plates and cultured at 5% CO_2_, 37 °C for 24, 48, and 72 h, respectively. The cell morphology in 24‐well plates was observed by optical microscope at the corresponding time points. Subsequently, each well was added with 1000 µL DMEM containing 100 µL CCK‐8 reagent, cultured at 37 °C for another 2 h and transferred into 96‐well plates, and the optical density values of OD_450_ at the corresponding time were detected by a microplate reader (Spark 10 m, TECAN).

In Vitro Antibacterial Activity Test of the PUPDU‐Cu Elastomer

The antibacterial activities of the PUPDU‐Cu elastomer against Gram‐negative *E. coli* and Gram‐positive *S. aureus* were evaluated in vitro. First, 0.2 g PUPDU‐Cu elastomer was sterilized and completely soaked in 2 mL bacterial suspension (10^6^ CFU mL^−1^) and co‐cultured for 20 h at 37 °C and 200 rpm. Then, the bacterial suspension after co‐cultured with the PUPDU‐Cu elastomer was extracted and the optical density at 600 nm (OD_600_) was detected to calculate the bactericidal viability. At the same time, 1 mL of the bacterial suspension after co‐cultured with the PUPDU‐Cu elastomer was serially diluted with sterile phosphate‐buffered saline (PBS, pH = 7.4). Then 100 µL 10^5^ times diluted bacterial suspension was evenly coated on the agar plates and incubated at 37 °C for 12 h. The antibacterial activity was tested three times for Gram‐negative *E. coli* and Gram‐positive *S. aureus*, respectively.

The bacterial activity was calculated by the following Equation (1):

(1) $$\begin{array}{ccc} \mathrm{bacterial}\;\mathrm{activity}\;\left(\%\right) & = & \mathrm{OD}\;\mathrm{of}\;\mathrm{experimental}\;\mathrm{group}/\\ & & \mathrm{OD}\;\mathrm{of}\;\mathrm{blank}\;\mathrm{group}\;\times \;100\;\left(\%\right) \end{array}$$

The bacterial ratio was calculated by the following Equation (2):

(2) $$\mathrm{bacterial}\;\mathrm{ratio}\;\left(\%\right)=1-\mathrm{bacterial}\;\mathrm{activity}\;\left(\%\right)$$

### Synthesis of MXene Nanosheets

The MXene (Ti_3_C_2_T_x_) nanosheets were obtained by selectively etching the monoatomic Al layers of MAX phase (Ti_3_AlC_2_) in LiF‐HCl etchant, as the previous work.^[^ ^3^ ^]^

### Fabrication of PUPDU‐Cu Elastomer Film with the Conductive MXene Nanosheets‐Coated Microdome Array Microstructures

First, 6 g PUPDU‐Cu elastomer was dissolved in 4 mL tetrahydrofuran (THF) to obtain PUPDU‐Cu gel. Next, the LLDPE film with a thickness of ≈5 µm was attached onto a through‐hole silicon mold on the tray of the spin‐coating machine. Then, open the vacuum button of the spin‐coating machine and drop the PUPDU‐Cu gel onto the surface of the LLDPE film with the concave microstructures for spin coating (1800 rpm, 90 s), and the curing of PUPDU‐Cu gel could be obtained under the rapid volatilization of the highly volatile THF during the spin coating process. Subsequently, the curing film was stripped from the LLDPE film to obtain a convex microdome array microstructure‐contained PUPDU‐Cu elastomer film with a thickness of 250 µm. Finally, the MXene nanosheets suspension (6 mg mL^−1^) was dropped onto the surface of the convex microdome array microstructure‐contained PUPDU‐Cu elastomer film and dried to obtain the PUPDU‐Cu elastomer film with the conductive MXene nanosheets‐coated microdome array microstructures.

### Fabrication of the Skin Bionic Multifunctional Flexible Electronic Sensor

First, the interdigitated electrode‐coated PUPDU‐Cu elastomer film was prepared by screen printing conductive silver paste with customized patterns onto the PUPDU‐Cu elastomer film with a thickness of 250 µm. Then, the PUPDU‐Cu elastomer film with the conductive MXene nanosheets‐coated microdome array microstructures and the interdigitated electrode‐coated PUPDU‐Cu elastomer film were attached together face‐to‐face.

### Characterization and Measurements

The chemical structure of the synthesized PUPDU‐Cu elastomer was investigated by the ^1^H NMR spectrometers (Bruker, Avance III HD 400 MHz), the ^13^C NMR (Bruker, Avance III HD 400 MHz) and the FTIR (Thermo Fisher, Nicolet 6700). The thermal performance of the PUPDU‐Cu elastomer was measured by the DSC (Mettler, TGA/DSC 3+) and the TGA (NETZSCH, TG209F1). The XPS spectra was obtained from X‐ray photoelectron spectrometer (Thermo, Escalab 250). The XRD spectra was obtained from X‐ray diffraction patterns (Rigaku, D/Max 2500). The sample microstructure and morphology were investigated by SEM (Hitachi, S‐4800), TEM (Hitachi, HT7700), AFM (Bruker, multimode8), and optical microscope (ZEISS, Axiolab 5). The mechanical properties of the PUPDU‐Cu elastomer were tested by using a universal testing machine (ESM303, MARK‐10) at a steady rate of 50 mm min^−1^ at room temperature, and the samples were cut into dumbbell shapes (5 mm (length) × 8 mm (width) × 1 mm (thickness)) using a customized cutter. The current variation of the sensor was real‐timely measured by a CHI660E electrochemical workstation. The real‐time wireless sensing of the sensor was achieved by connecting a wireless transmitter (FLUKE 3000 FC). The manipulator and somatosensory mechanical glove were designed by Hangzhou Zhongling Technology Co., Ltd. The informed written consent from all participants was obtained for the human activity experiments prior to the research.

## Conflict of Interest

The authors declare no conflict of interest.

## Author Contributions

Investigation, Resources, Data Curation, Writing‐Original Draft, K.L.; Conceptualization, Methodology, Supervision, P.W.; Writing‐Review & Editing, P.W.; Experimental results in Figures S25, S29, and S30 (Supporting Information), M.W.; Finite‐element simulation, C.H.; Validation, Software, M.W., C.H., Y.Y., and Y.N.; Resources, Validation, L.Z.

## Supporting information

Supporting Information

## Data Availability

The data that support the findings of this study are available from the corresponding author upon reasonable request.

## References

[1] H. C. Ates , P. Q. Nguyen , L. Gonzalez‐Macia , E. Morales‐Narváez , F. Güder , J. J. Collins , C. Dincer , Nat. Rev. Mater. 2022, 7, 887. 35910814 10.1038/s41578-022-00460-x PMC9306444

[2] Z. Cui , W. Wang , H. Xia , C. Wang , J. Tu , S. Ji , J. M. R. Tan , Z. Liu , F. Zhang , W. Li , Z. Lv , Z. Li , W. Guo , N. Y. Koh , K. B. Ng , X. Feng , Y. Zheng , X. Chen , Adv. Mater. 2022, 34, 2207016. 10.1002/adma.202207016 36134530

[3] L. Wang , N. Li , Y. Zhang , P. Di , M. Li , M. Lu , K. Liu , Z. Li , J. Ren , L. Zhang , P. Wan , Matter 2022, 5, 3417.

[4] S. Pyo , J. Lee , K. Bae , S. Sim , J. Kim , Adv. Mater. 2021, 33, 2005902. 10.1002/adma.202005902 33887803

[5] J. Kim , A. S. Campbell , B. E.‐F. De Ávila , J. Wang , Nat. Biotechnol. 2019, 37, 389. 30804534 10.1038/s41587-019-0045-y PMC8183422

[6] M.‐H. Seo , J.‐Y. Yoo , S.‐Y. Choi , J.‐S. Lee , K.‐W. Choi , C. K. Jeong , K. J. Lee , J.‐B. Yoon , ACS Nano 2017, 11, 1520. 28135071 10.1021/acsnano.6b06842

[7] H. Kong , Z. Song , W. Li , Y. Bao , D. Qu , Y. Ma , Z. Liu , W. Wang , Z. Wang , D. Han , L. Niu , ACS Nano 2021, 15, 16218. 34605628 10.1021/acsnano.1c05199

[8] B. Ji , Q. Zhou , B. Hu , J. Zhong , J. Zhou , B. Zhou , Adv. Mater. 2021, 33, 2100859. 10.1002/adma.202100859 34062019

[9] N. Bai , L. Wang , Y. Xue , Y. Wang , X. Hou , G. Li , Y. Zhang , M. Cai , L. Zhao , F. Guan , X. Wei , C. F. Guo , ACS Nano 2022, 16, 4338. 35234457 10.1021/acsnano.1c10535

[10] S. R. A. Ruth , V. R. Feig , H. Tran , Z. Bao , Adv. Funct. Mater. 2020, 30, 2003491.

[11] Z. Shi , L. Meng , X. Shi , H. Li , J. Zhang , Q. Sun , X. Liu , J. Chen , S. Liu , Nano‐Micro Lett. 2022, 14, 141. 10.1007/s40820-022-00874-w PMC9256895 35789444

[12] K. Cao , M. Wu , J. Bai , Z. Wen , J. Zhang , T. Wang , M. Peng , T. Liu , Z. Jia , Z. Liang , L. Jiang , Adv. Funct. Mater. 2022, 32, 2202360.

[13] J. Wang , B. Wu , P. Wei , S. Sun , P. Wu , Nat. Commun. 2022, 13, 4411. 35906238 10.1038/s41467-022-32140-3 PMC9338060

[14] S. Liu , Y. Rao , H. Jang , P. Tan , N. Lu , Matter 2022, 5, 1104.

[15] Y. Lee , J. Park , A. Choe , S. Cho , J. Kim , H. Ko , Adv. Funct. Mater. 2020, 30, 1904523.

[16] T. Someya , M. Amagai , Nat. Biotechnol. 2019, 37, 382. 30940942 10.1038/s41587-019-0079-1

[17] Y. Pang , K. Zhang , Z. Yang , S. Jiang , Z. Ju , Y. Li , X. Wang , D. Wang , M. Jian , Y. Zhang , R. Liang , H. Tian , Y. Yang , T.‐L. Ren , ACS Nano 2018, 12, 2346. 29378401 10.1021/acsnano.7b07613

[18] Y.‐E. Shin , Y.‐J. Park , S. K. Ghosh , Y. Lee , J. Park , H. Ko , Adv. Sci. 2022, 9, 2105423. 10.1002/advs.202105423 PMC8948547 35072354

[19] A. Vahidmohammadi , J. Rosen , Y. Gogotsi , Science 2021, 372, 1165. 10.1126/science.abf1581 34112665

[20] Y. Wei , P. Zhang , R. A. Soomro , Q. Zhu , B. Xu , Adv. Mater. 2021, 33, 2103148. 10.1002/adma.202103148 34423479

[21] D. Wang , C. Zhou , A. S. Filatov , W. Cho , F. Lagunas , M. Wang , S. Vaikuntanathan , C. Liu , R. F. Klie , D. V. Talapin , Science 2023, 379, 1242. 36952427 10.1126/science.add9204

[22] D. Lei , N. Liu , T. Su , Q. Zhang , L. Wang , Z. Ren , Y. Gao , Adv. Mater. 2022, 34, 2110608. 10.1002/adma.202110608 35291047

[23] Y. Zhang , M. Gong , P. Wan , Matter 2021, 4, 2655.

[24] Y. Wang , T. Guo , E. Alhajji , Z. Tian , Z. Shi , Y.‐Z. Zhang , H. N. Alshareef , Adv. Energy Mater. 2023, 13, 2202860.

[25] S. Pu , Z. Wang , Y. Xie , J. Fan , Z. Xu , Y. Wang , H. He , X. Zhang , W. Yang , H. Zhang , Adv. Funct. Mater. 2023, 33, 2208715.

[26] H. Zhou , S. J. Han , H.‐D. Lee , D. Zhang , M. Anayee , S. H. Jo , Y. Gogotsi , T.‐W. Lee , Adv. Mater. 2022, 34, 2206377. 10.1002/adma.202206377 36037306

[27] G. Yang , F. Liu , J. Zhao , L. Fu , Y. Gu , L. Qu , C. Zhu , J.‐J. Zhu , Y. Lin , Coord. Chem. Rev. 2023, 479, 215002.

[28] N. Wu , Y. Yang , C. Wang , Q. Wu , F. Pan , R. Zhang , J. Liu , Z. Zeng , Adv. Mater. 2023, 35, 2207969. 10.1002/adma.202207969 36281792

[29] Y. Zhang , X. Chen , C. Luo , J. Gu , M. Li , M. Chao , X. Chen , T. Chen , L. Yan , X. Wang , Adv. Funct. Mater. 2022, 32, 2111660.

[30] Y. Gao , L. Yu , J. C. Yeo , C. T. Lim , Adv. Mater. 2020, 32, 1902133. 10.1002/adma.201902133 31339200

[31] H. Liu , K. Sun , X.‐L. Guo , Z.‐L. Liu , Y.‐H. Wang , Y. Yang , D. Yu , Y.‐T. Li , T.‐L. Ren , ACS Nano 2022, 16, 21527. 36449370 10.1021/acsnano.2c10342

[32] W. Li , M. Xu , J. Gao , X. Zhang , H. Huang , R. Zhao , X. Zhu , Y. Yang , L. Luo , M. Chen , H. Ji , L. Zheng , X. Wang , W. Huang , Adv. Mater. 2023, 35, 2207447. 10.1002/adma.202207447 36353895

[33] X. Wang , S. Zhan , Z. Lu , J. Li , X. Yang , Y. Qiao , Y. Men , J. Sun , Adv. Mater. 2020, 32, 2005759. 10.1002/adma.202005759 33175420

[34] J. Ekeocha , C. Ellingford , M. Pan , A. M. Wemyss , C. Bowen , C. Wan , Adv. Mater. 2021, 33, 2008052. 10.1002/adma.202008052 34165832

[35] J. Chen , Q. Peng , X. Peng , H. Zhang , H. Zeng , Chem. Rev. 2022, 122, 14594. 36054924 10.1021/acs.chemrev.2c00215

[36] N. Zheng , Y. Xu , Q. Zhao , T. Xie , Chem. Rev. 2021, 121, 1716. 33393759 10.1021/acs.chemrev.0c00938

[37] B. Li , P.‐F. Cao , T. Saito , A. P. Sokolov , Chem. Rev. 2023, 123, 701. 36577085 10.1021/acs.chemrev.2c00575

[38] S. Wang , M. W. Urban , Nat. Rev. Mater. 2020, 5, 562.

[39] L. Zhang , Z. Liu , X. Wu , Q. Guan , S. Chen , L. Sun , Y. Guo , S. Wang , J. Song , E. M. Jeffries , C. He , F.‐L. Qing , X. Bao , Z. You , Adv. Mater. 2019, 31, 1901402. 10.1002/adma.201901402 30977571

[40] Y. Zhou , L. Li , Z. Han , Q. Li , J. He , Q. Wang , Chem. Rev. 2023, 123, 558. 36260027 10.1021/acs.chemrev.2c00231

[41] C. Liu , J.‐T. Kim , D. S. Yang , D. Cho , S. Yoo , S. R. Madhvapathy , H. Jeong , T. Yang , H. Luan , R. Avila , J. Park , Y. Wu , K. Bryant , M. Cho , J. Lee , J. Y. Kwak , W. Ryu , Y. Huang , R. G. Nuzzo , J. A. Rogers , Adv. Funct. Mater. 2023, 33, 2302256.

[42] X. Xun , Z. Zhang , X. Zhao , B. Zhao , F. Gao , Z. Kang , Q. Liao , Y. Zhang , ACS Nano 2020, 14, 9066. 32658455 10.1021/acsnano.0c04158

[43] J. Xiong , G. Thangavel , J. Wang , X. Zhou , P. S. Lee , Sci. Adv. 2020, 6, eabb4246. 32832644 10.1126/sciadv.abb4246 PMC7439505

[44] X. Zhu , W. Zhang , G. Lu , H. Zhao , L. Wang , ACS Nano 2022, 16, 16724. 36215403 10.1021/acsnano.2c06264

[45] M. Zhu , Y. Wang , M. Lou , J. Yu , Z. Li , B. Ding , Nano Energy 2021, 81,105669.

[46] X. Peng , K. Dong , C. Ye , Y. Jiang , S. Zhai , R. Cheng , D. Liu , X. Gao , J. Wang , Z. L. Wang , Sci. Adv. 2020, 6, eaba9624. 32637619 10.1126/sciadv.aba9624 PMC7319766

[47] M. Godoy‐Gallardo , U. Eckhard , L. M. Delgado , Y. J. D. de Roo Puente , M. Hoyos‐Nogues , F. J. Gil , R. A. Perez , Bioact. Mater. 2021, 6, 4470. 34027235 10.1016/j.bioactmat.2021.04.033 PMC8131399

[48] M. L. Ermini , V. Voliani , ACS Nano 2021, 15, 6008. 33792292 10.1021/acsnano.0c10756 PMC8155324

[49] J. Zhou , H. Liu , Y. Sun , C. Wang , K. Chen , Adv. Funct. Mater. 2021, 31, 2011133.

[50] N. Tang , R. Zhang , Y. Zheng , J. Wang , M. Khatib , X. Jiang , C. Zhou , R. Omar , W. Saliba , W. Wu , M. Yuan , D. Cui , H. Haick , Adv. Mater. 2022, 34, 2106842. 10.1002/adma.202106842 34741350

[51] N. Li , J. Peng , W.‐J. Ong , T. Ma , P. Z. Arramel , J. Jiang , X. Yuan , C. Zhang , Matter 2021, 4, 377.

[52] M. Lu , C. Huang , Z. Xu , Y. Yuan , M. Wang , M. Xiao , L. Zhang , P. Wan , Adv. Funct. Mater. 2023, 33, 2306591.

[53] K. Meng , X. Xiao , W. Wei , G. Chen , A. Nashalian , S. Shen , X. Xiao , J. Chen , Adv. Mater. 2022, 34, 2109357. 10.1002/adma.202109357 35044014

[54] X. Wang , Z. Feng , Y. Xia , G. Zhang , L. Wang , L. Chen , Y. Wu , J. Yang , Z. L. Wang , Nano Energy 2022, 102, 107710.

[55] Y. Zhang , Z. Xu , Y. Yuan , C. Liu , M. Zhang , L. Zhang , P. Wan , Adv. Funct. Mater. 2023, 33, 2300299.

[56] Y. Zhao , W. Gao , K. Dai , S. Wang , Z. Yuan , J. Li , W. Zhai , G. Zheng , C. Pan , C. Liu , C. Shen , Adv. Mater. 2021, 33, 2102332. 10.1002/adma.202102332 34554616

[57] C. Pang , J. H. Koo , A. Nguyen , J. M. Caves , M.‐G. Kim , A. Chortos , K. Kim , P. J. Wang , J. B.‐H. Tok , Z. Bao , Adv. Mater. 2015, 27, 634. 25358966 10.1002/adma.201403807

[58] S. Min , D. H. Kim , D. J. Joe , B. W. Kim , Y. H. Jung , J. H. Lee , B.‐Y. Lee , I. l Doh , J. An , Y.‐N. Youn , B. Joung , C. D. Yoo , H.‐S. Ahn , K. J. Lee , Adv. Mater. 2023, 35, 2301627. 10.1002/adma.202301627 36960816

[59] R. Yin , D. Wang , S. Zhao , Z. Lou , G. Shen , Adv. Funct. Mater. 2021, 31, 2008936.

[60] W. Heng , S. Solomon , W. Gao , Adv. Mater. 2022, 34, 2107902. 10.1002/adma.202107902 PMC9035141 34897836

