# Supplementary material for: Flexible Bioinspired Healable Antibacterial Electronics for Intelligent Human‐Machine Interaction Sensing
Source: Adv Sci (Weinh). 2023 Dec 22;11(10):2305672. doi: 10.1002/advs.202305672 (PMC10933681; doi:10.1002/advs.202305672)
Supplement: Supplementary file 1 — Supporting Information [file ADVS-11-2305672-s001.pdf]

ADVANCED SCIENCE
Open Access

Supporting Information

for *Adv. Sci.*, DOI 10.1002/advs.202305672

Flexible Bioinspired Healable Antibacterial Electronics for Intelligent Human-Machine Interaction Sensing

*Kuo Liu, Mingcheng Wang, Chenlin Huang, Yue Yuan, Yao Ning, Liqun Zhang and Pengbo Wan**

Copyright WILEY-VCH Verlag GmbH & Co. KGaA, 69469 Weinheim, Germany, 2021.

Supporting Information

Flexible Bioinspired Healable Antibacterial Electronics for Intelligent Human-Machine Interaction Sensing

Kuo Liu, Mingcheng Wang, Chenlin Huang, Yue Yuan, Yao Ning, Liqun Zhang, and Pengbo Wan*

K. Liu, M. C. Wang, C. L. Huang, Y. Yuan, Y. Ning, Prof. L. Q. Zhang, Prof. P. B. Wan
College of Materials Science and Engineering, State Key Laboratory of Organic-Inorganic Composites, Beijing University of Chemical Technology, Beijing 100029, China
Email: pbwan@mail.buct.edu.cn

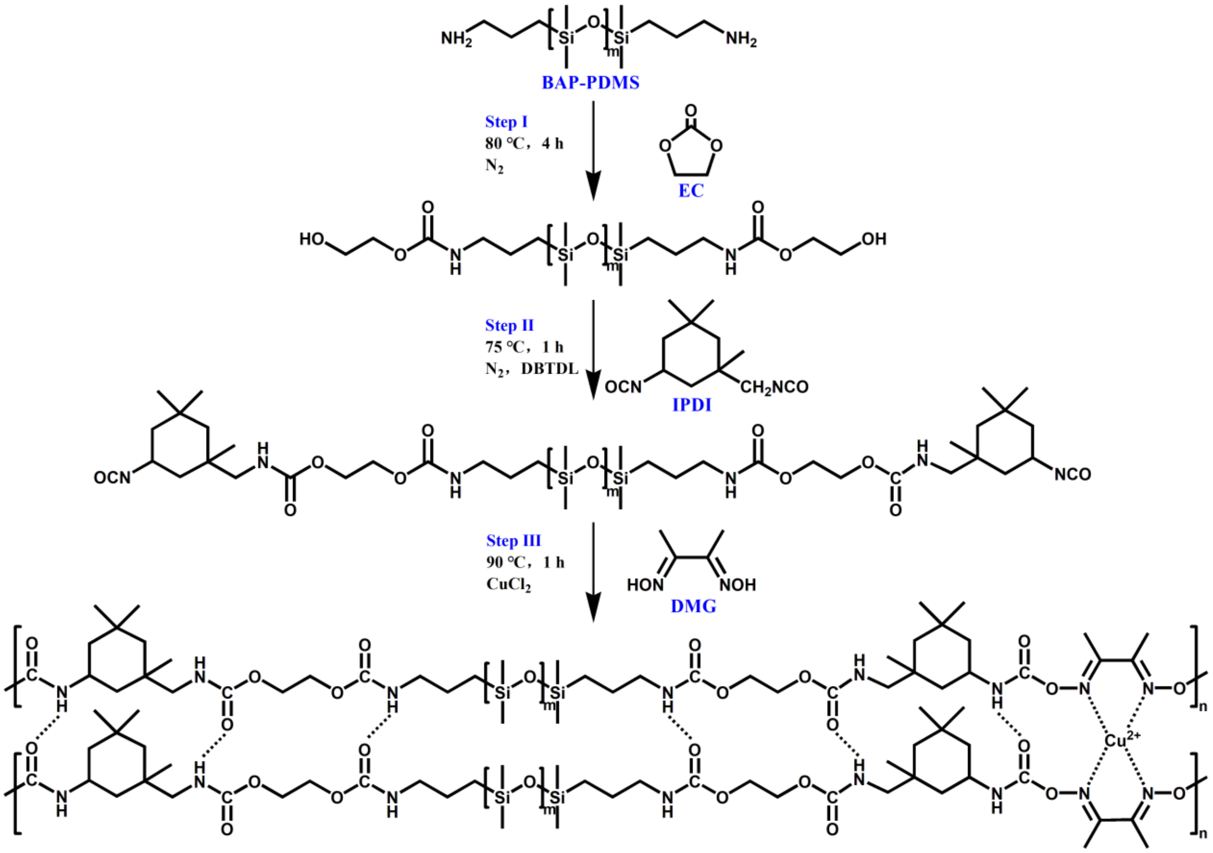

Figure S1. Synthesis procedure of PUPDU-Cu elastomer.

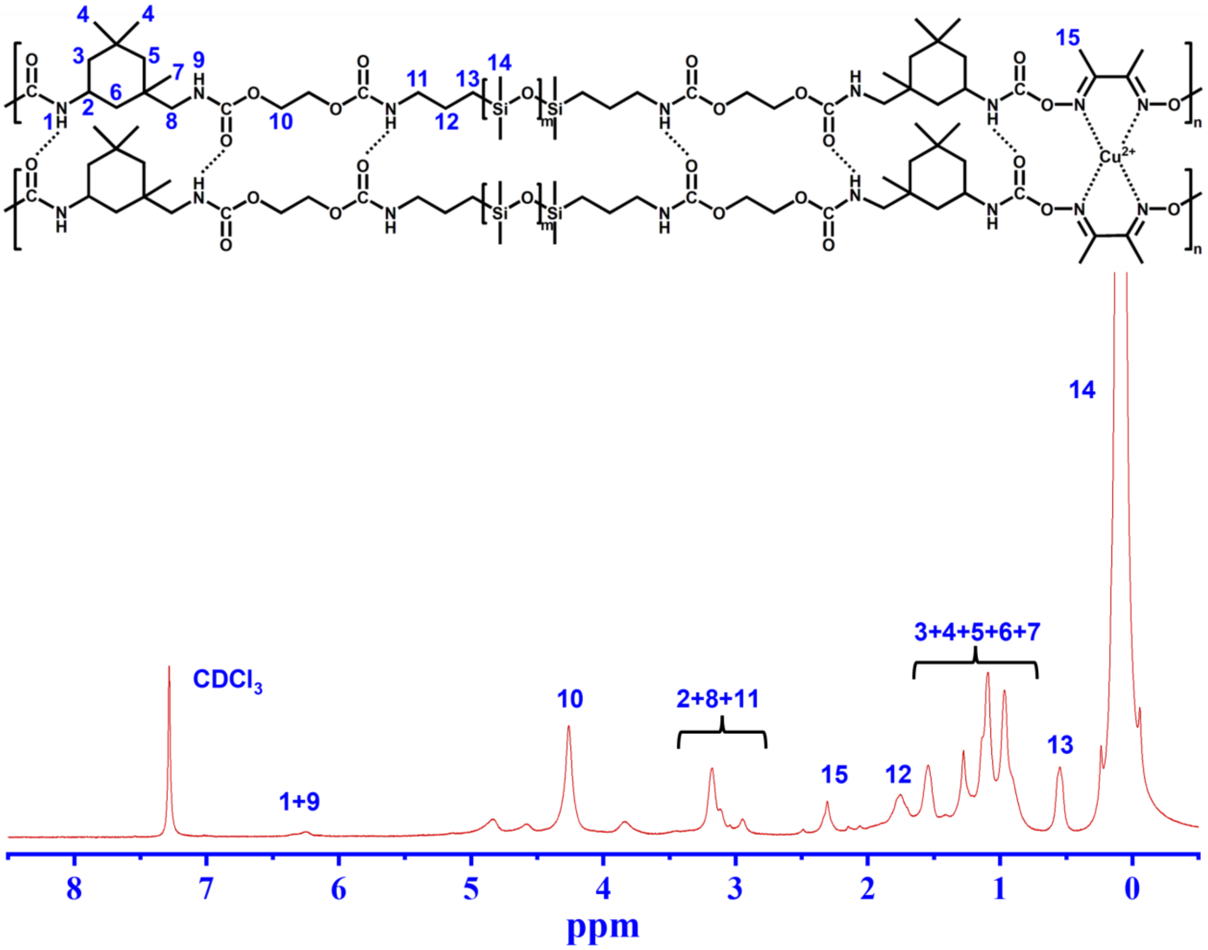

Figure S2. ^{1}H NMR spectrum of the PUPDU-Cu elastomer (deuterated $CDCl_3$).

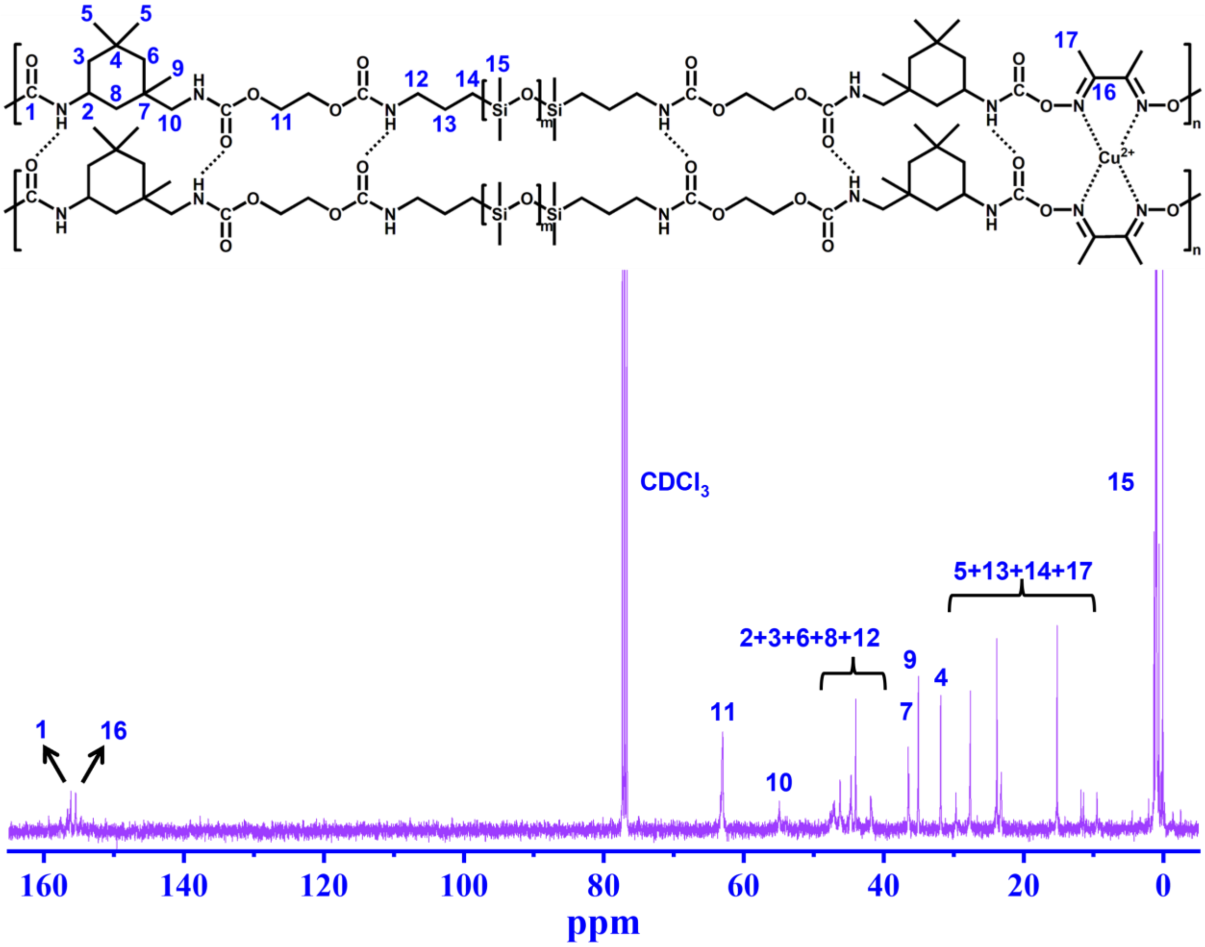

Figure S3. ^{13}C NMR spectrum of the PUPDU-Cu elastomer (deuterated $CDCl_3$).

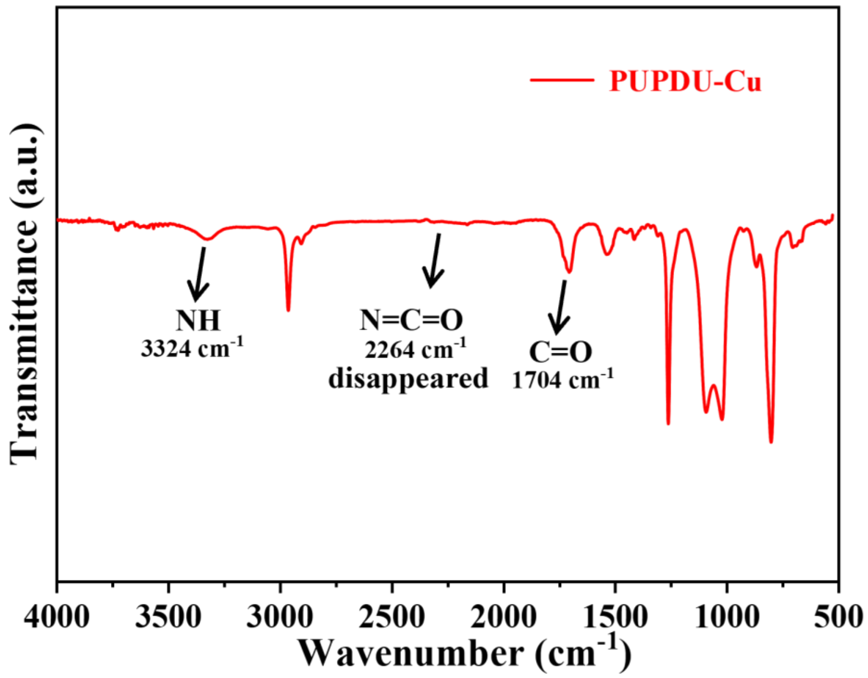

Figure S4. FTIR spectra of the PUPDU-Cu elastomer.

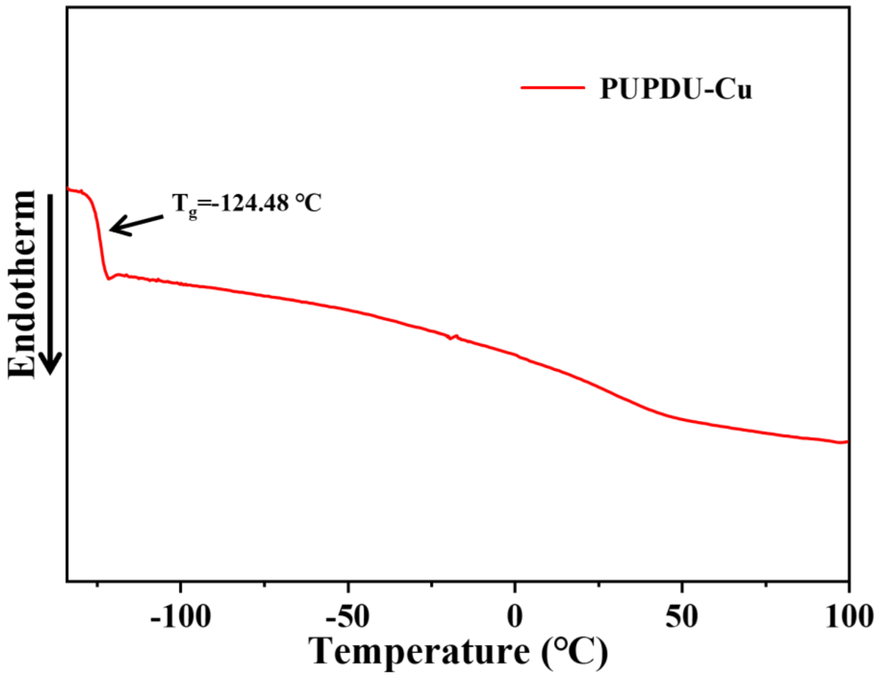

Figure S5. DSC curve of the PUPDU-Cu elastomer.

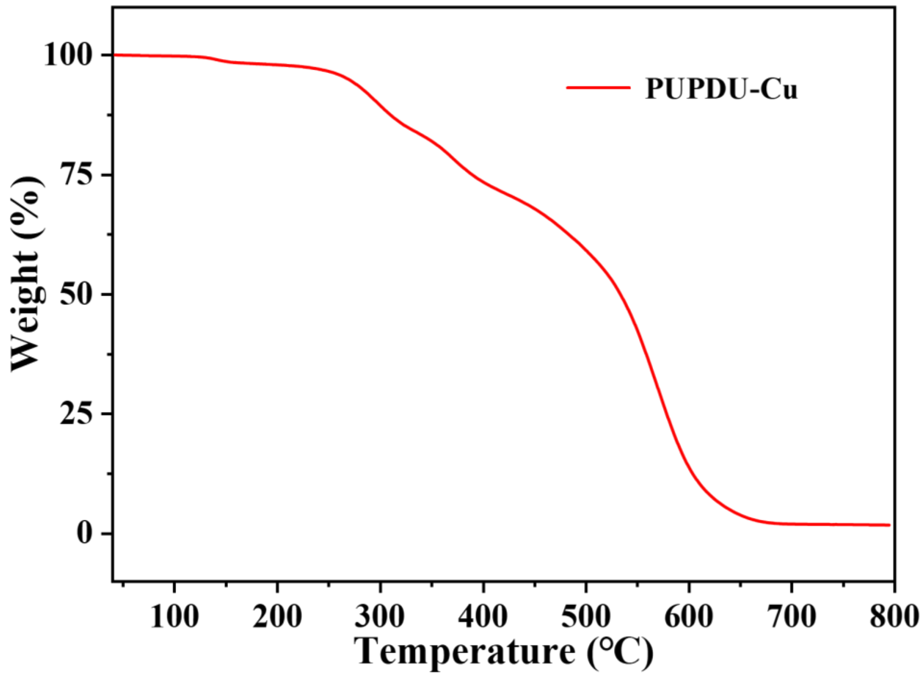

Figure S6. TGA curve of the PUPDU-Cu elastomer.

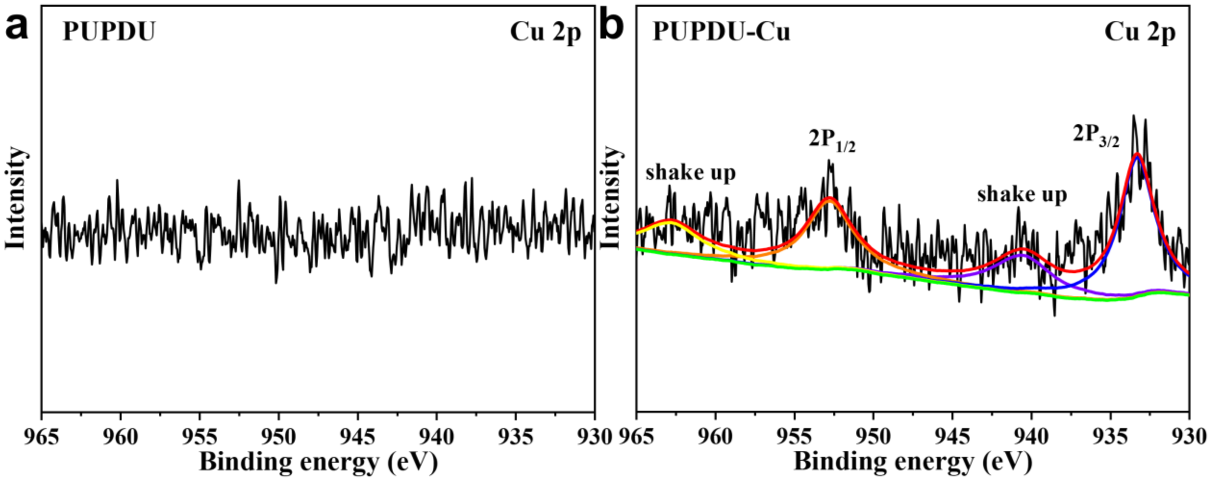

Figure S7. XPS spectra of the PUPDU elastomer and the PUPDU-Cu elastomer. (a) High-resolution Cu 2p spectra of the PUPDU elastomer. (b) High-resolution Cu 2p spectra of the PUPDU-Cu elastomer.

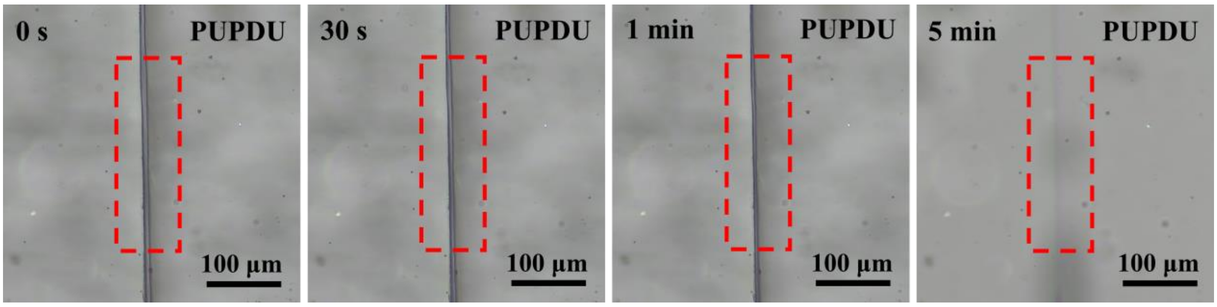

Figure S8. Optical microscope images of the healing of the scratch of the PUPDU elastomer film, showing a rapid healing, and the scratch almost disappeared gradually after 5 min at 60°C.

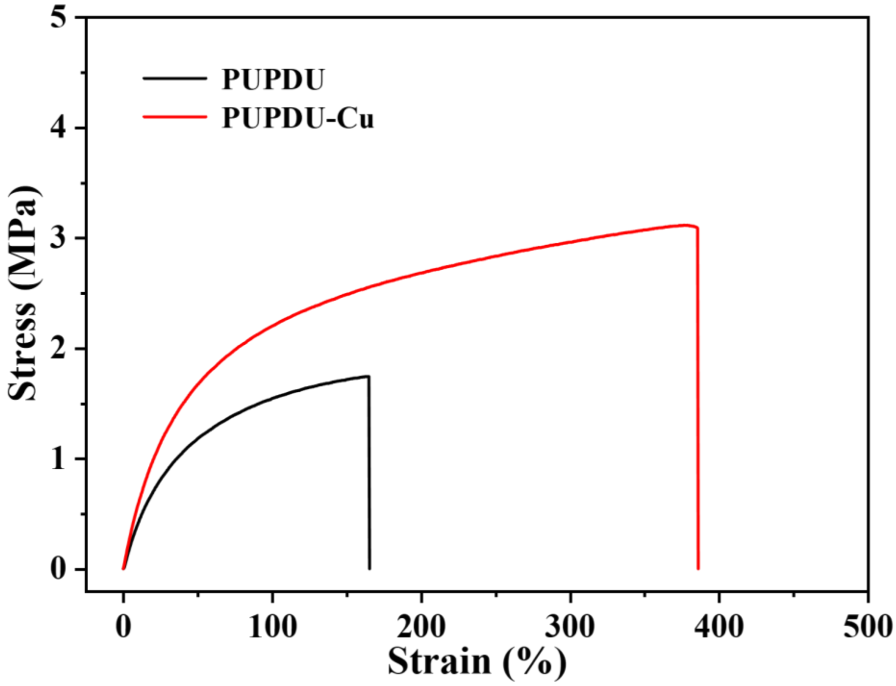

Figure S9. The tensile stress-strain curves of the PUPDU elastomer and the PUPDU-Cu elastomer.

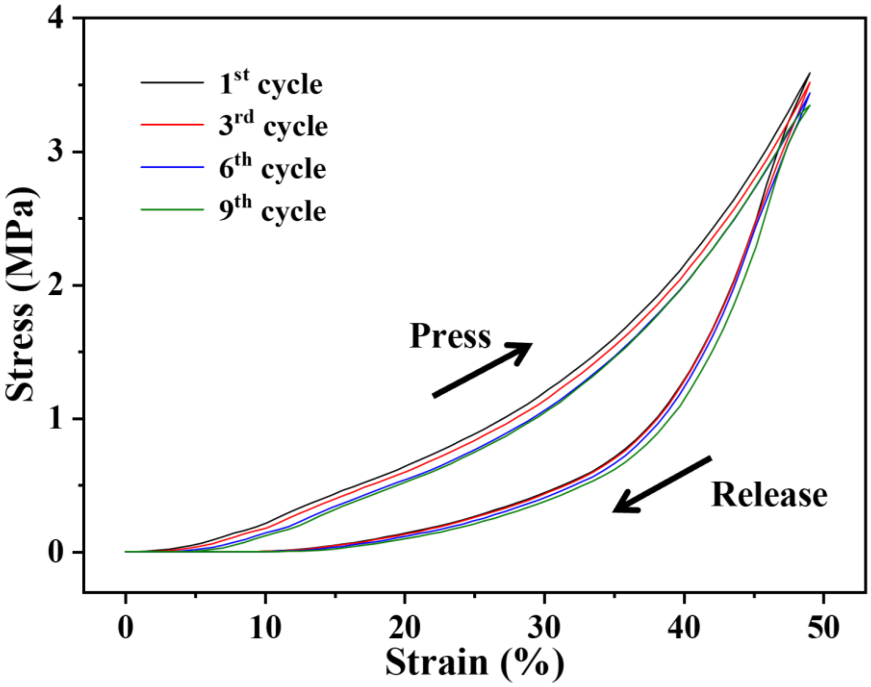

Figure S10. The compressive stress-strain curves of the PUPDU-Cu elastomer.

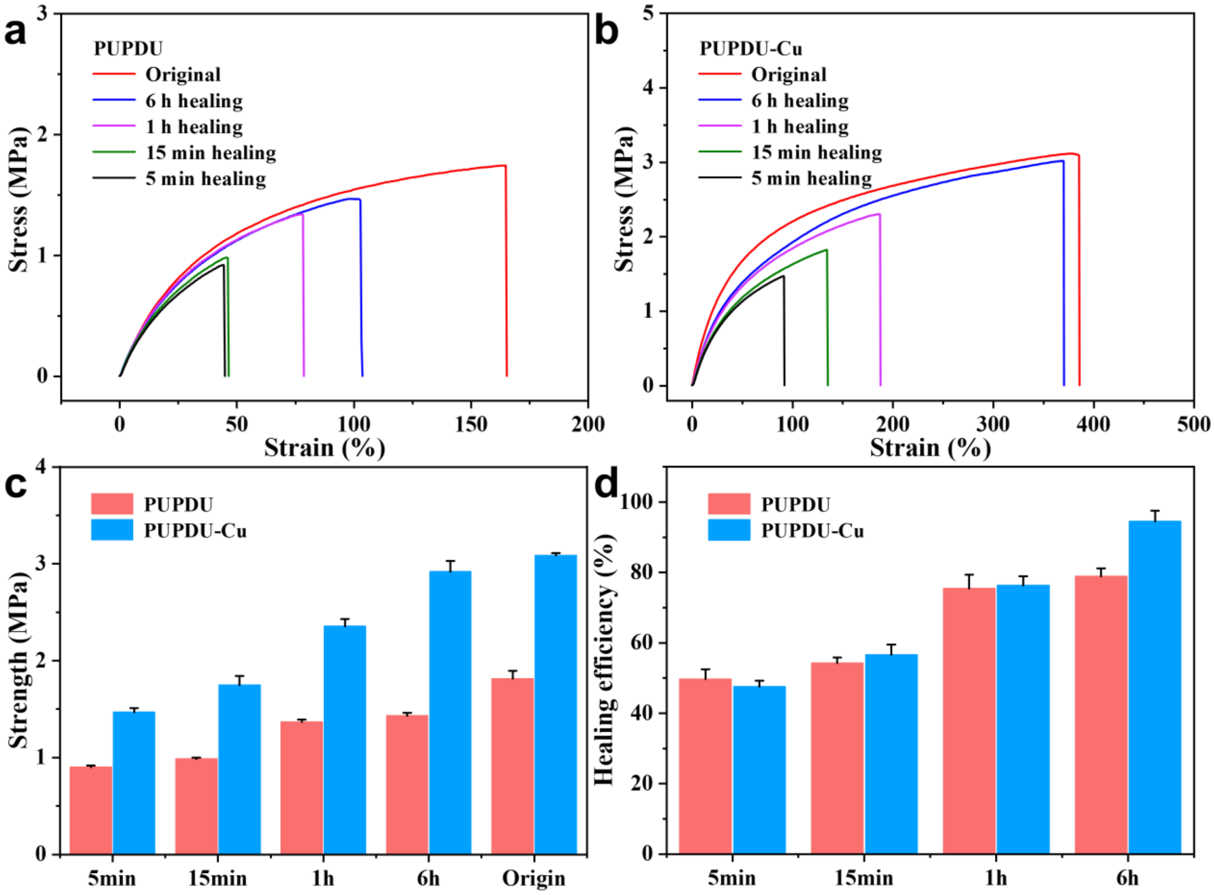

Figure S11. (a-b) The tensile stress-strain curves of the dumbbell-shaped PUPDU elastomer spline and the dumbbell-shaped PUPDU-Cu elastomer before and after cutting, and after healing at 60°C for 5 min, 15 min, 1 h and 6 h respectively (the Original curve in Figure S11b is the same as the PUPDU-Cu curve in Figure S9). (c) The healing strength and (d) the healing efficiency of the PUPDU elastomer and the PUPDU-Cu elastomer after healing at 60°C for 5 min, 15 min, 1 h and 6 h respectively.

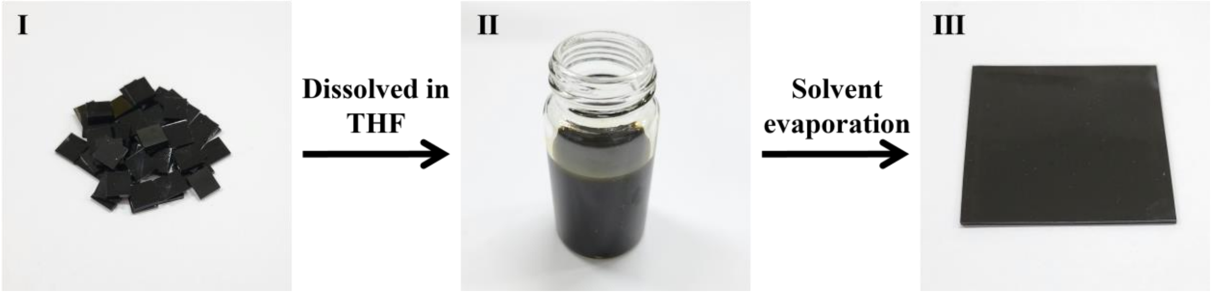

Figure S12. Recycling process of the PUPDU-Cu elastomer. (I) Photograph of the PUPDU-Cu elastomer film after cutting into fragments. (II) Photograph of the PUPDU-Cu elastomer fragments dissolved in THF. (III) Photograph of the obtained recycled PUPDU-Cu elastomer film.

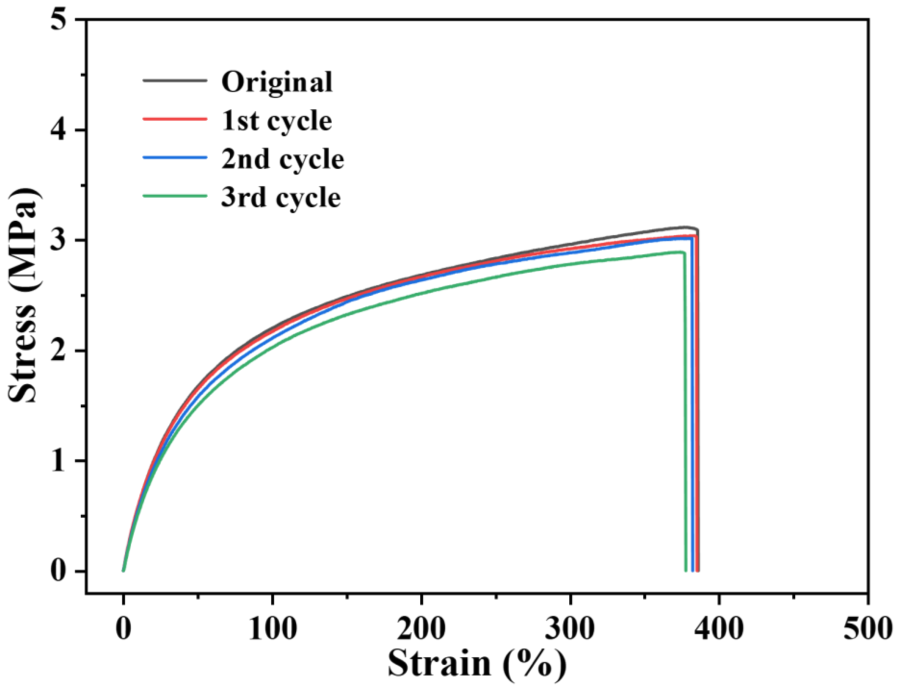

Figure S13. Tensile stress-strain curves of the PUPDU-Cu elastomer with different recovery times (the Original curve in Figure S13 is the same as the Original curve in Figure S11b).

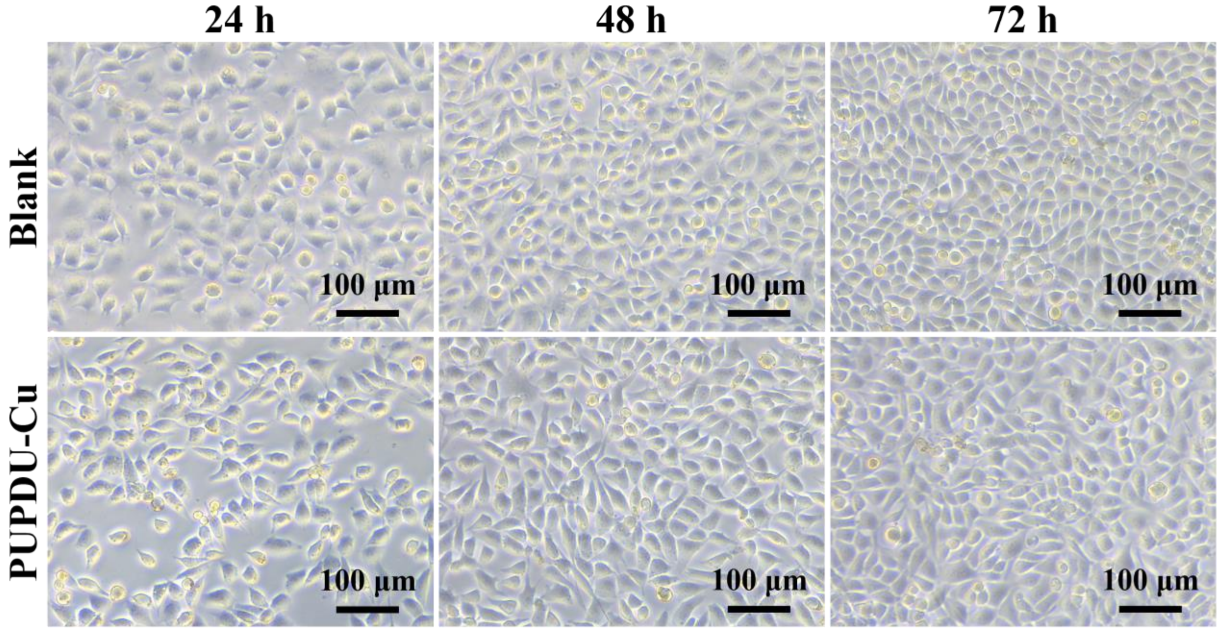

Figure S14. Optical microscope images of L929 cells after cultured with the PUPDU-Cu elastomer extract for 24 h, 48 h, and 72 h, respectively.

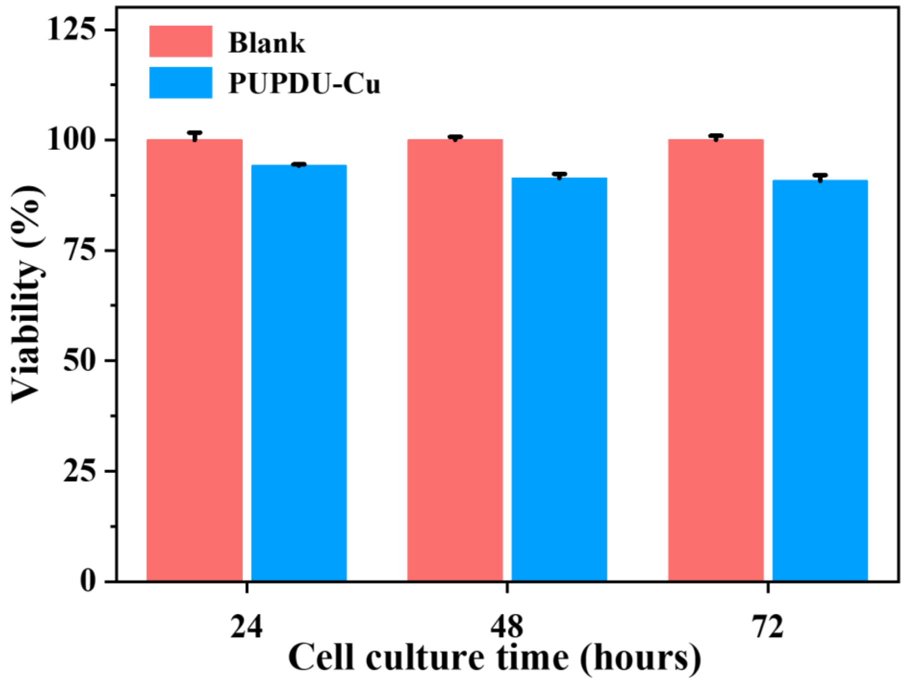

Figure S15. Cell viability of L929 cells after cultured with the PUPDU-Cu elastomer extract for 24 h, 48 h, and 72 h, respectively.

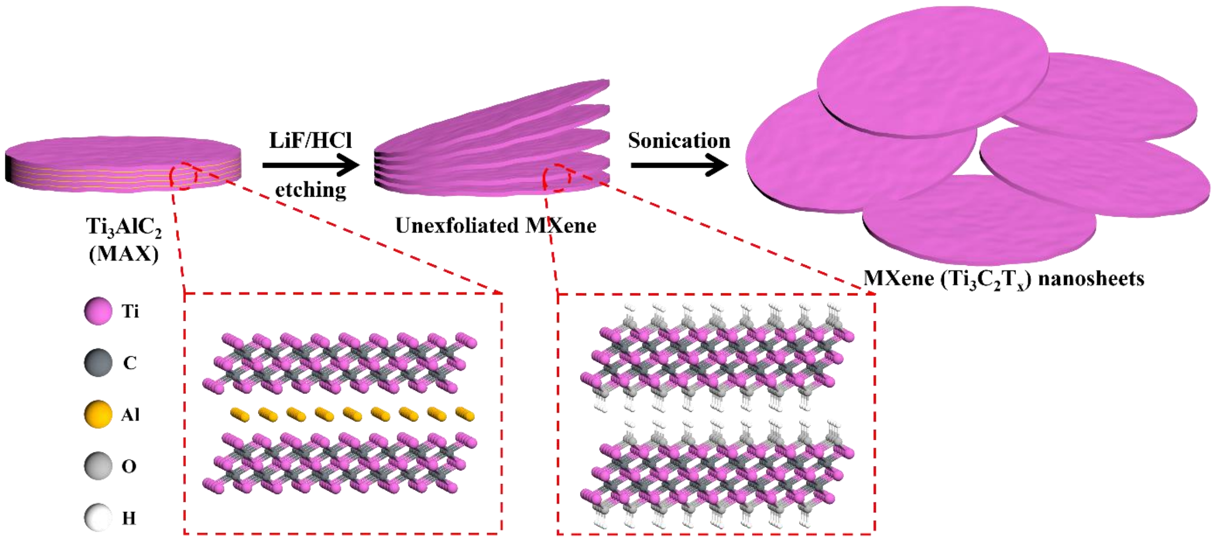

Figure S16. Synthesis schematic of MXene nanosheets.

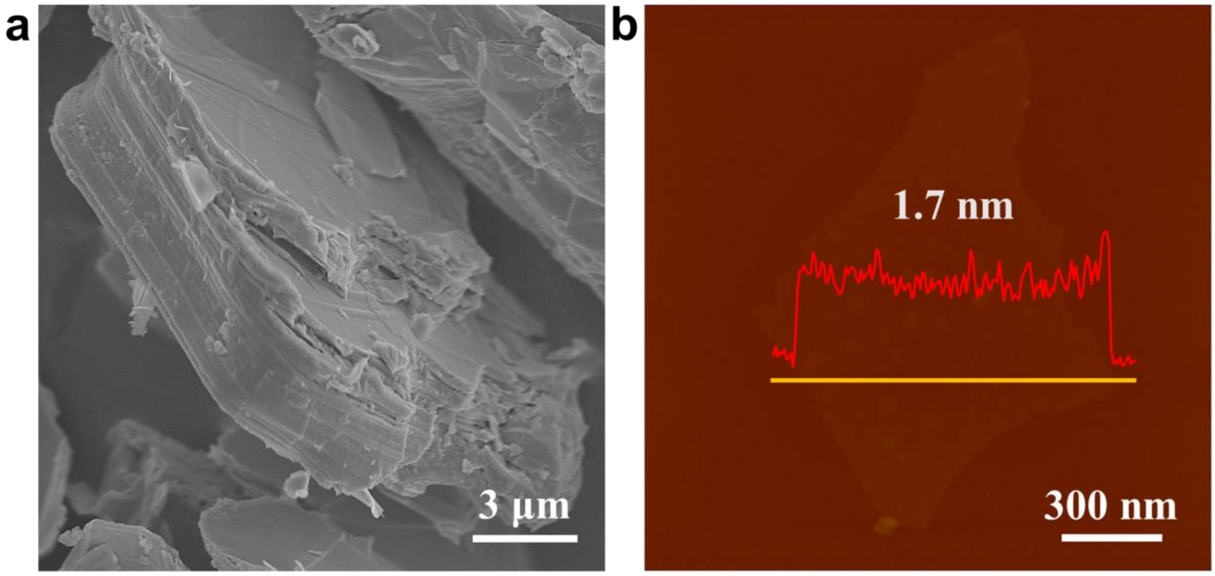

Figure S17. (a) SEM image of the MAX phase (Ti_3AlC_2). (b) AFM image of the MXene nanosheet deposited on a silicon wafer.

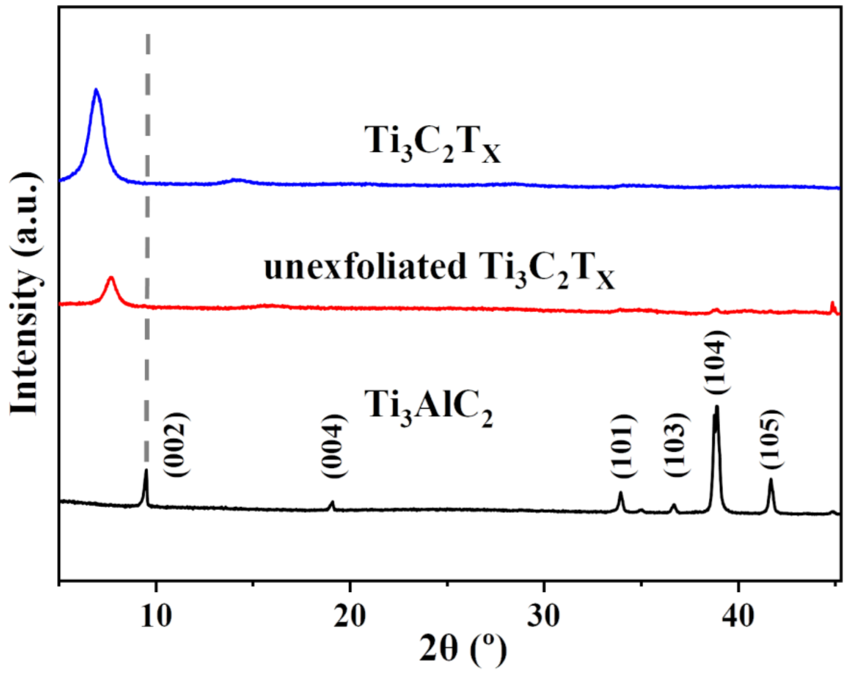

Figure S18. XRD patterns of the Ti_3AlC_2, the unexfoliated MXene and the MXene nanosheet.

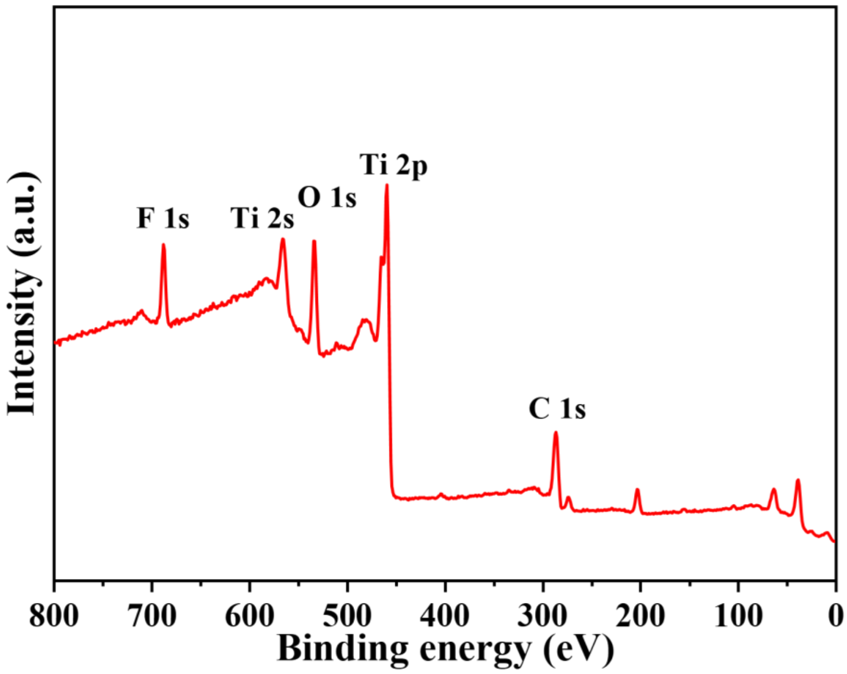

Figure S19. XPS spectrum of the MXene nanosheet.

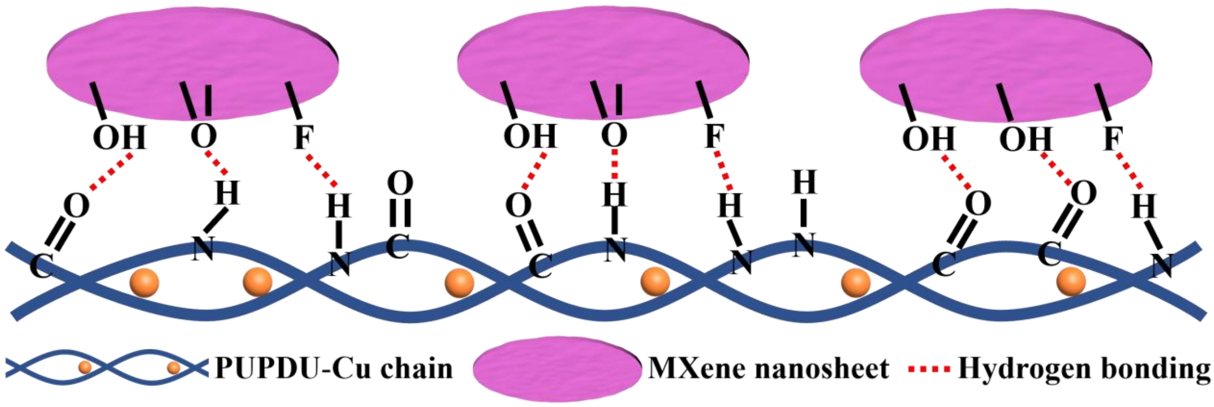

Figure S20. Hydrogen bonding between MXene nanosheets and the PUPDU-Cu polymer chain.

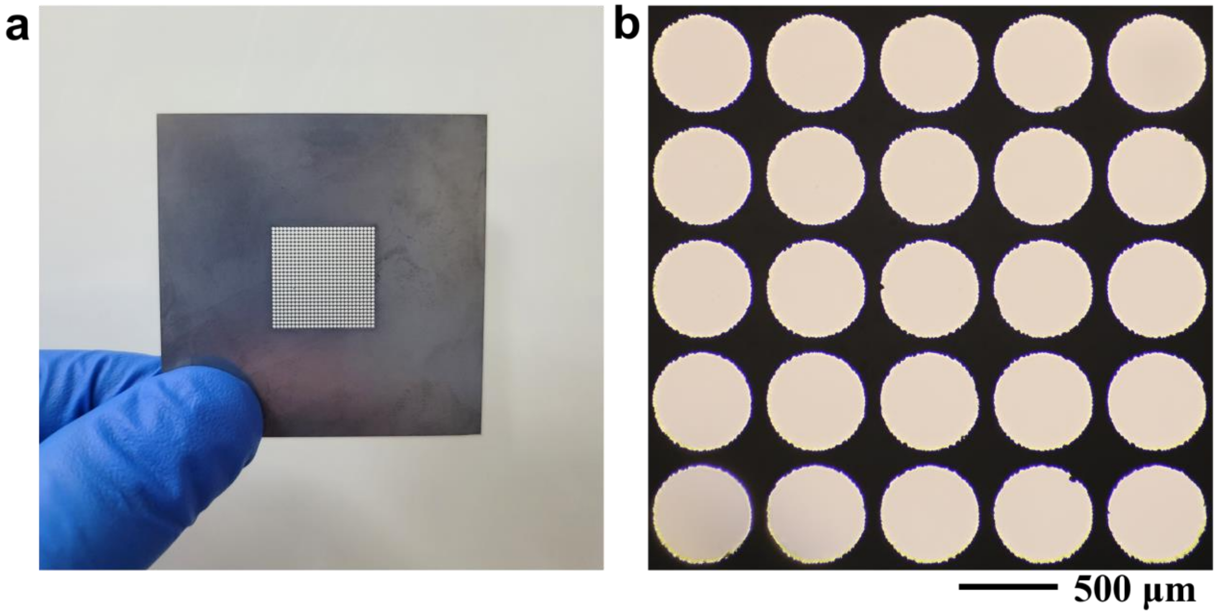

Figure S21. (a) Optical image of the through-hole silicon mold. The area of the through hole on the silicon mold is about 1.7 cm × 1.7 cm. (b) Optical microscope image of the through-hole silicon mold. The diameter of each hole is 500 µm, and the distance between adjacent holes is about 70 µm.

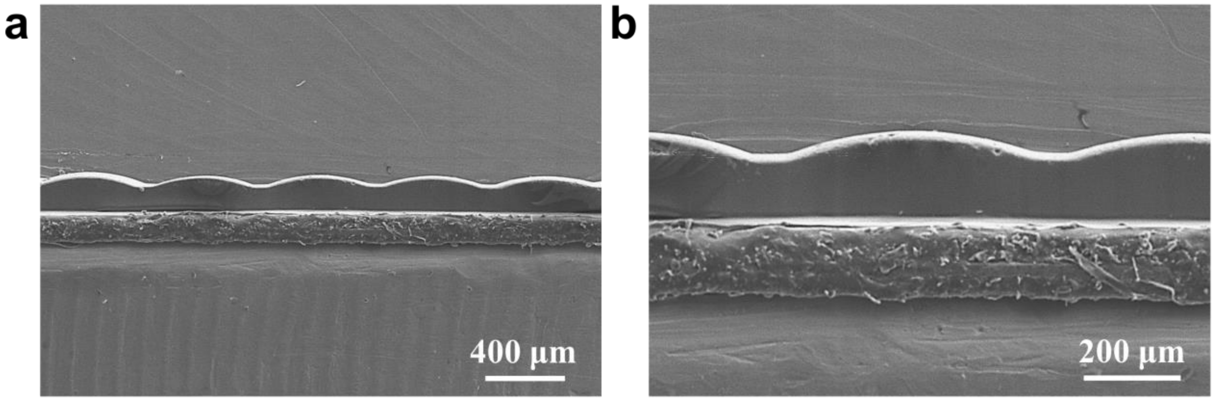

Figure S22. SEM images of the cross-section of the microdome array microstructures coated with conductive MXene nanosheets.

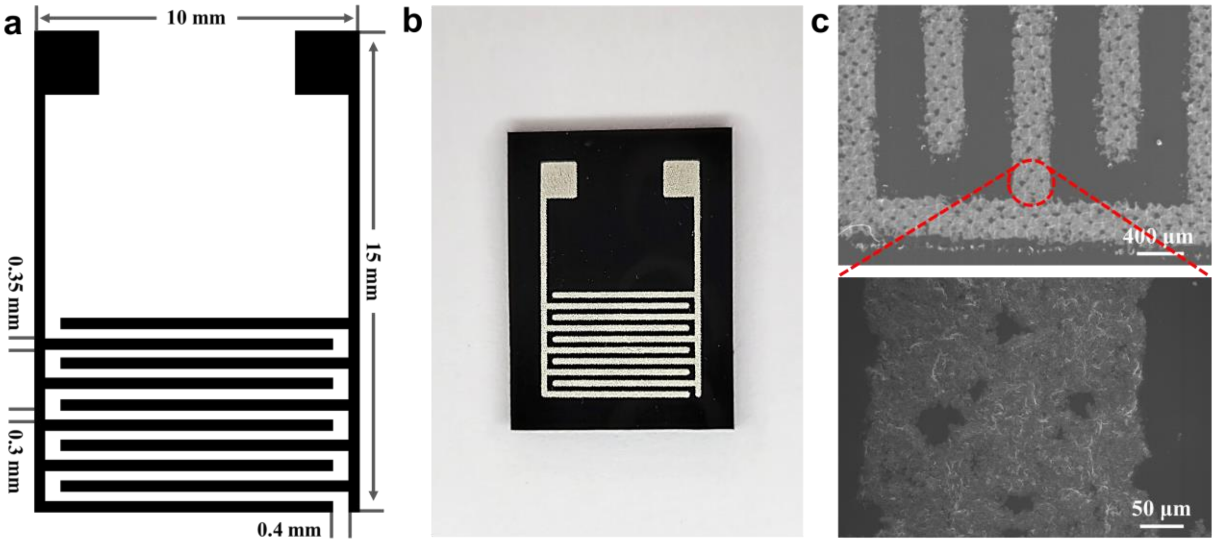

Figure S23. (a) Schematic diagram of the dimension of the interdigitated electrode. (b) Optical photograph of the PUPDU-Cu elastomer film with the printed interdigitated electrode. (c) SEM images of the interdigitated electrode.

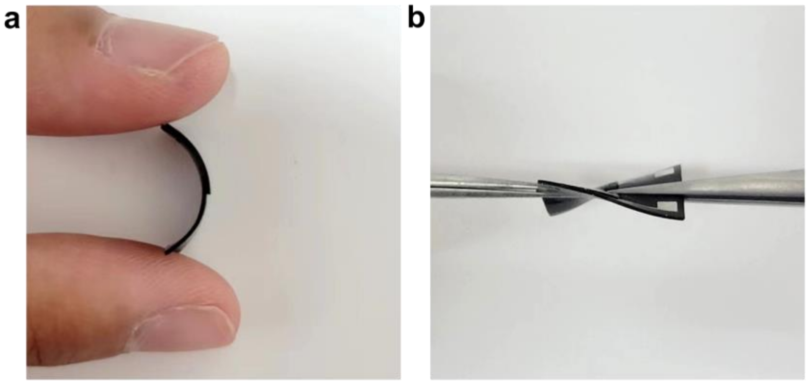

Figure S24. Photographs for demonstrating (a) the bending and (b) the twisting of the flexible electronic sensors.

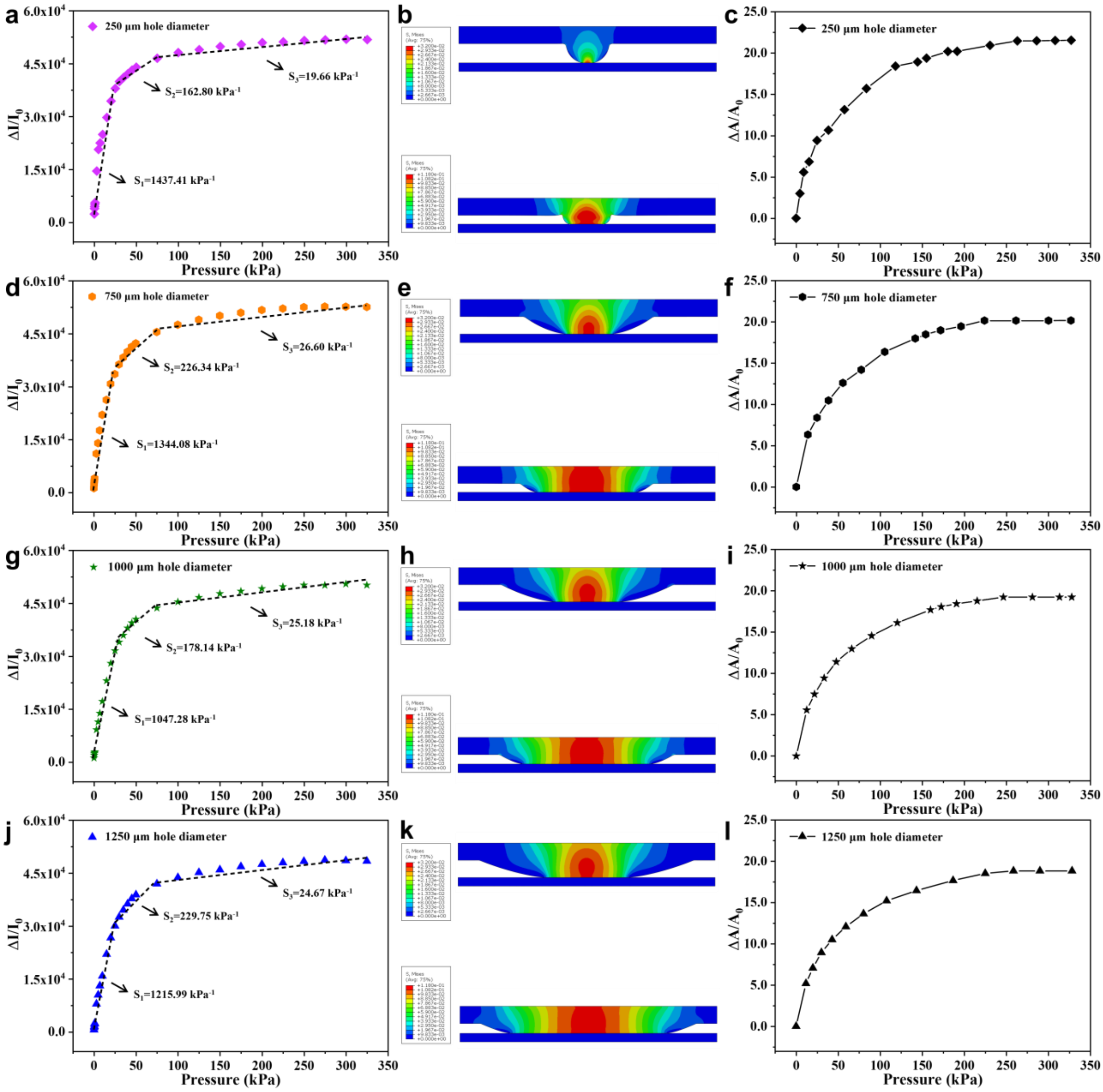

Figure S25. The sensing performances and the simulated results of the flexible electronic sensors from the different hole diameters of the through-hole silicon molds. (a) The sensing performance of the flexible electronic sensor prepared from the through-hole silicon mold with 250 μm hole diameter, (b) the corresponding finite-element simulation of stress distribution of microdome structure (250 μm hole diameter of the through-hole silicon mold) under the pressures of 32 and 118 kPa respectively, and (c) the relative area change ($\Delta A/A_0$) between the microdome structure (250 μm hole diameter of the through-hole silicon mold) and the flat electrode under different pressures calculated by finite-element simulation. (d) The sensing performance of the flexible electronic sensor prepared from the through-hole silicon mold with 750 μm hole diameter, (e) the corresponding finite-element simulation of stress distribution of microdome structure (750 μm hole diameter of the through-hole silicon mold) under the pressures of 32 and 118 kPa respectively, and (f) the relative area change ($\Delta A/A_0$) between the microdome structure (750 μm hole diameter of the through-hole silicon mold) and the flat electrode under different pressures calculated by finite-element simulation. (g) The sensing performance of the flexible electronic sensor prepared from the through-hole silicon mold with 750 μm hole diameter, (h) the corresponding finite-element simulation of stress distribution of microdome structure (750 μm hole diameter of the

through-hole silicon mold) under the pressures of 32 and 18 kPa respectively, and (i) the relative area change ($\Delta A/A_0$) between the microdome structure (750 μm hole diameter of the through-hole silicon mold) and the flat electrode under different pressures calculated by finite-element simulation. (j) The sensing performance of the flexible electronic sensor prepared from the through-hole silicon mold with 750 μm hole diameter, (k) the corresponding finite-element simulation of stress distribution of microdome structure (750 μm hole diameter of the through-hole silicon mold) under the pressures of 32 and 118 kPa respectively, and (l) the relative area change ($\Delta A/A_0$) between the microdome structure (750 μm hole diameter of the through-hole silicon mold) and the flat electrode under different pressures calculated by finite-element simulation.

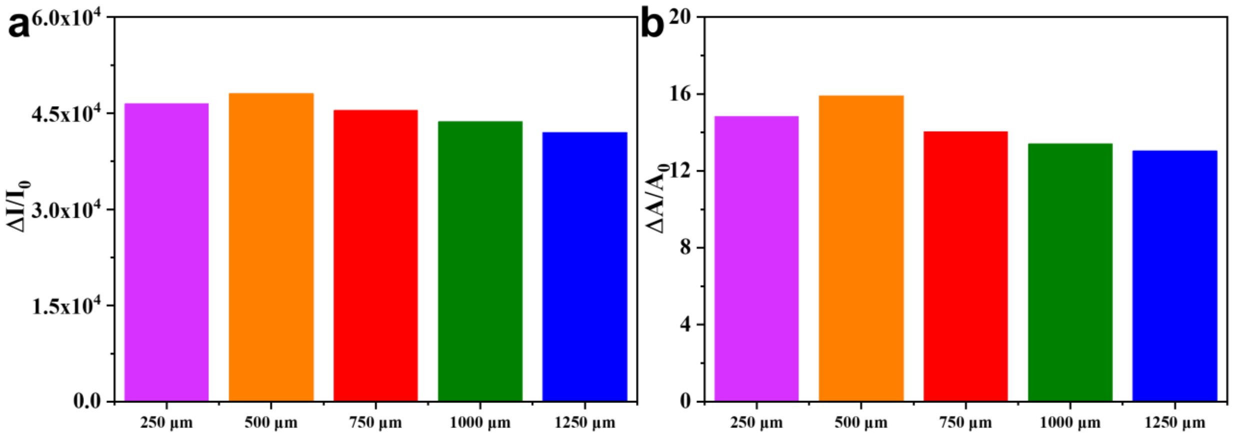

Figure S26. (a) The experimental sensing response ($\Delta I/I_0$) of the flexible electronic sensors from the different hole diameters of the through-hole silicon molds under external pressure of 75 kPa, obtained from Figure S25a, S25d, S25g, S25j and Figure 4c. (b) The relative area change ($\Delta A/A_0$) between microdome structures with different diameters and the contact electrode under external pressure of 75 kPa calculated by finite-element simulation from Figure S25c, S25f, S25i, S25l and Figure 4f.

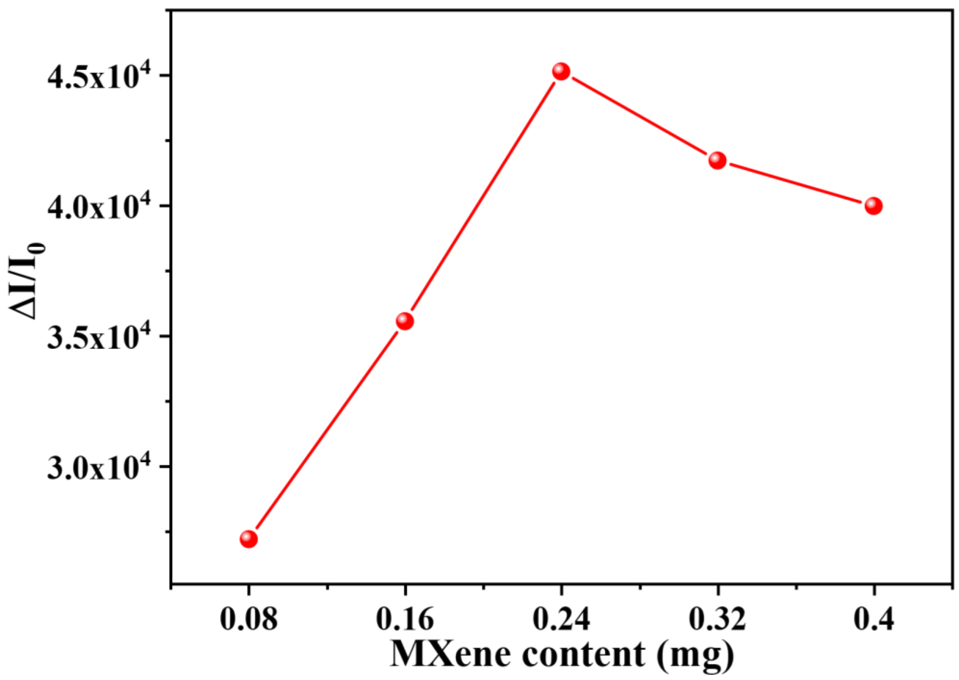

Figure S27. The sensing response variations of the flexible electronic sensor with different amounts of MXene nanosheets coating under external pressure of 50 kPa.

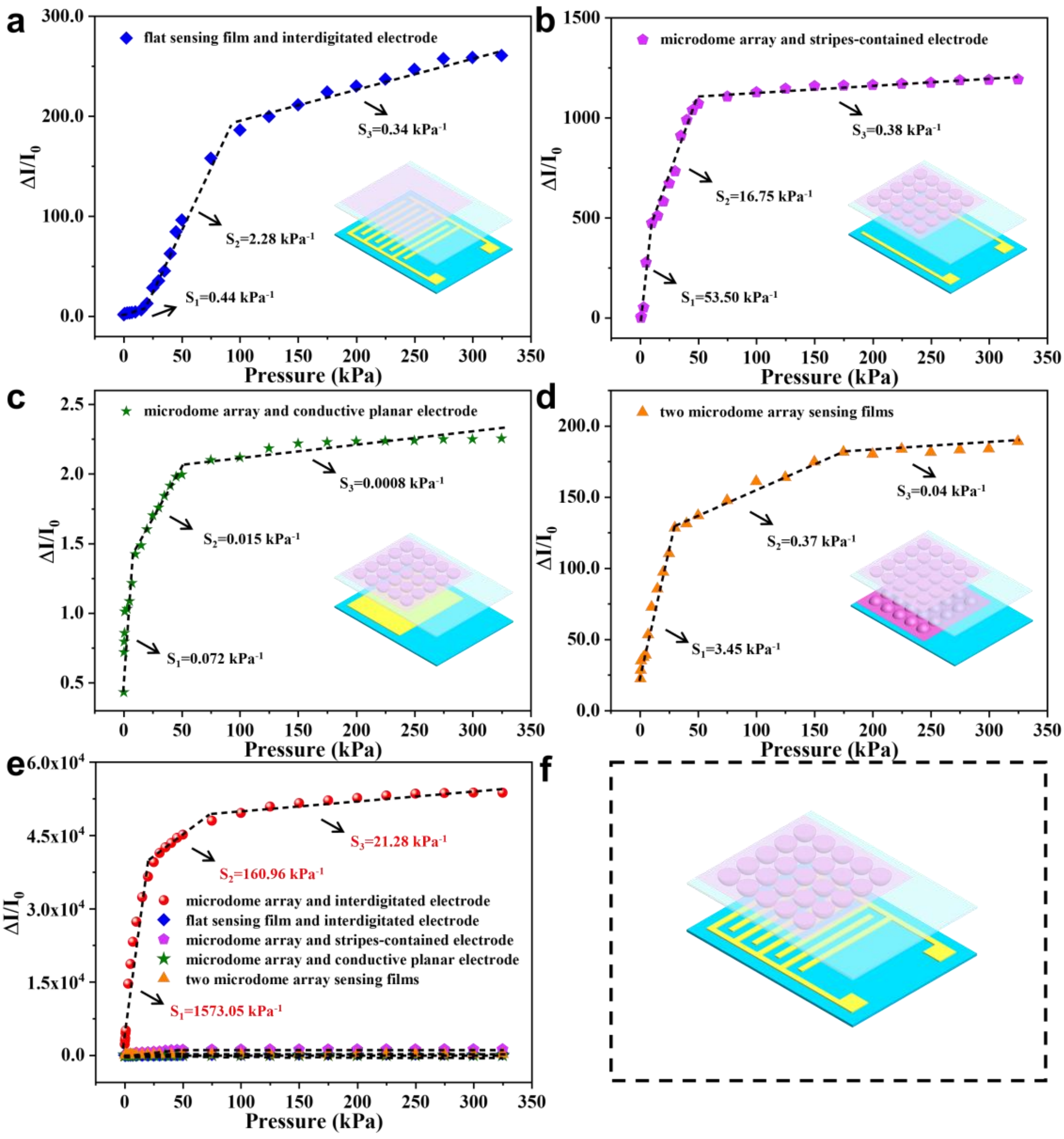

Figure S28. The sensing performances of the flexible electronic sensors assembled with different sensing layers and different electrodes. (a) The sensing performance of the flexible electronic sensor assembled from the MXene nanosheets-coated flat PUPDU-Cu elastomer film and the interdigitated electrode-coated PUPDU-Cu elastomer film (Inset: the schematic of the corresponding as-assembled sensor). (b) The sensing performance of the flexible electronic sensor assembled from the PUPDU-Cu elastomer film with the conductive MXene nanosheets-coated microdome array microstructures and the two conductive stripes-contained electrode (Inset: the schematic of the corresponding as-assembled sensor). (c) The sensing performance of the flexible electronic sensor assembled from the PUPDU-Cu elastomer film with the conductive MXene nanosheets-coated microdome array microstructures and the conductive planar electrode (Inset: the schematic of the corresponding as-assembled sensor). (d) The sensing performance of the flexible electronic sensor assembled from two PUPDU-Cu elastomer films with the conductive MXene nanosheets-coated microdome array microstructures (Inset: the schematic of the corresponding as-assembled sensor). (e) The sensing performance comparison of the above three sensors with that of (f) the flexible electronic sensor assembled face-to-face from a PUPDU-Cu elastomer film with the conductive MXene nanosheets-coated microdome array microstructures and an interdigitated electrode-coated PUPDU-Cu elastomer film (*the sensing performance curves collected from Figure S28abcd and Figure 4c respectively*).

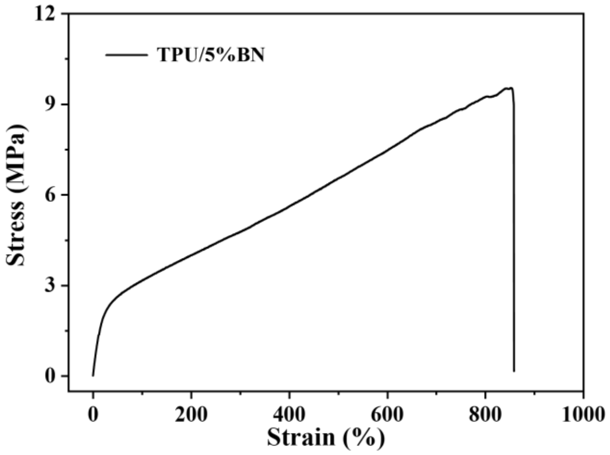

Figure S29. The tensile stress-strain curves of the TPU/5%BN elastomer.

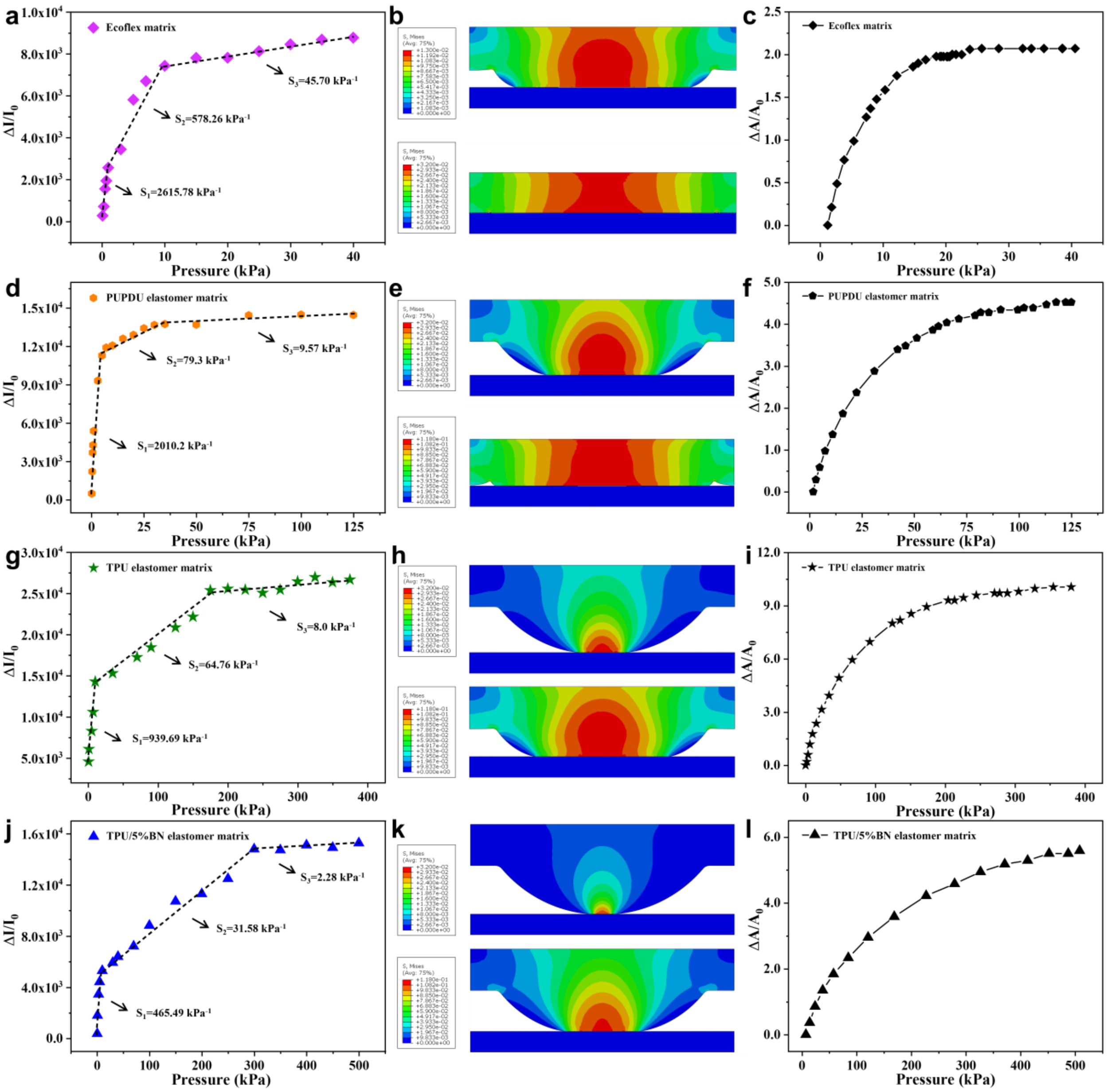

Figure S30. The sensing performances and the simulated results of the flexible electronic sensors prepared from different elastomer matrixes with variable moduli. (a) The sensing performance of the skin bionic flexible electronic sensor prepared from Ecoflex matrix with ~0.044 MPa[1]. (b) The finite-element simulation of stress distribution of microdome structure film prepared from Ecoflex matrix under the pressures of 13 and 32 kPa respectively. (c) The relative area change ($\Delta A/A_0$) between the microdome structure film prepared from Ecoflex matrix and the flat electrode of the sensor based on Ecoflex matrix under different pressures calculated by finite-element simulation. (d) The sensing performance of the skin bionic flexible electronic sensor prepared from PUPDU elastomer matrix with ~2.24 MPa. (e) The finite-element simulation of stress distribution of microdome structure film prepared from PUPDU elastomer matrix under the pressures of 32 and 118 kPa respectively. (f) The relative area change ($\Delta A/A_0$) between the microdome structure film prepared from PUPDU elastomer matrix and the flat electrode of the sensor based on PUPDU elastomer matrix under different pressures calculated by finite-element simulation. (g) The sensing performance of the skin bionic flexible electronic sensor prepared from TPU elastomer matrix with ~3.90 MPa[1]. (h) The finite-element simulation of stress distribution of microdome structure film

prepared from TPU elastomer matrix under the pressures of 32 and 118 kPa respectively. (i) The relative area change ($\Delta A/A_0$) between the microdome structure film prepared from TPU elastomer matrix and the flat electrode of the sensor based on TPU elastomer matrix under different pressures calculated by finite-element simulation. (j) The sensing performance of the skin bionic flexible electronic sensor prepared from TPU/5%BN elastomer matrix with ~4.60 MPa. (k) The finite-element simulation of stress distribution of microdome structure film prepared from TPU/5%BN elastomer matrix under the pressures of 32 and 118 kPa respectively. (l) The relative area change ($\Delta A/A_0$) between the microdome structure film prepared from TPU/5%BN elastomer matrix and the flat electrode of the sensor based on TPU/5%BN elastomer matrix under different pressures calculated by finite-element simulation.

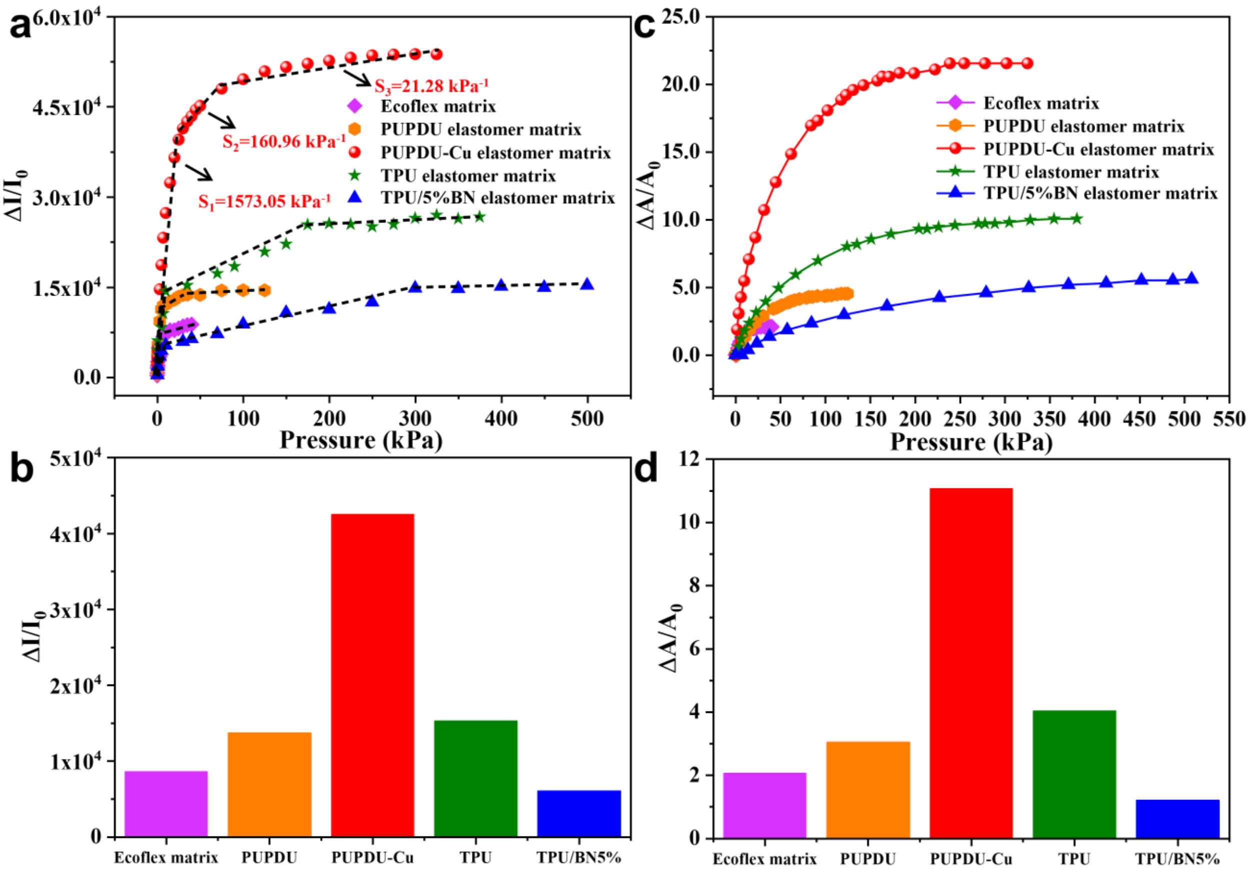

Figure S31. (a) The sensing performances comparison of the flexible electronic sensors (Figure S30adgj and Figure 4c) prepared from different elastomer matrixes with variable moduli (*the sensing performance curves collected from Figure S30adgj and Figure 4c*). (b) The experimental sensing response ($\Delta I/I_0$) of the flexible electronic sensors (Figure S30adgj and Figure 4c) prepared from different elastomer matrixes with variable moduli under external pressure of 35 kPa, obtained from Figure S31a, Figure S30adgj and Figure 4c. (c) The relative area change ($\Delta A/A_0$) comparison of the flexible electronic sensors between the microdome structures and the flat electrode (Figure S30cfil and Figure 4f) prepared from different elastomer matrixes with variable moduli by finite-element simulation (*the curves collected from Figure S30cfil and Figure 4f*). (d) The relative area change ($\Delta A/A_0$) comparison of the flexible electronic sensors between the microdome structures and the flat electrode under external pressure of 35 kPa calculated by finite-element simulation from Figure S31c, Figure S30cfil and Figure 4f.

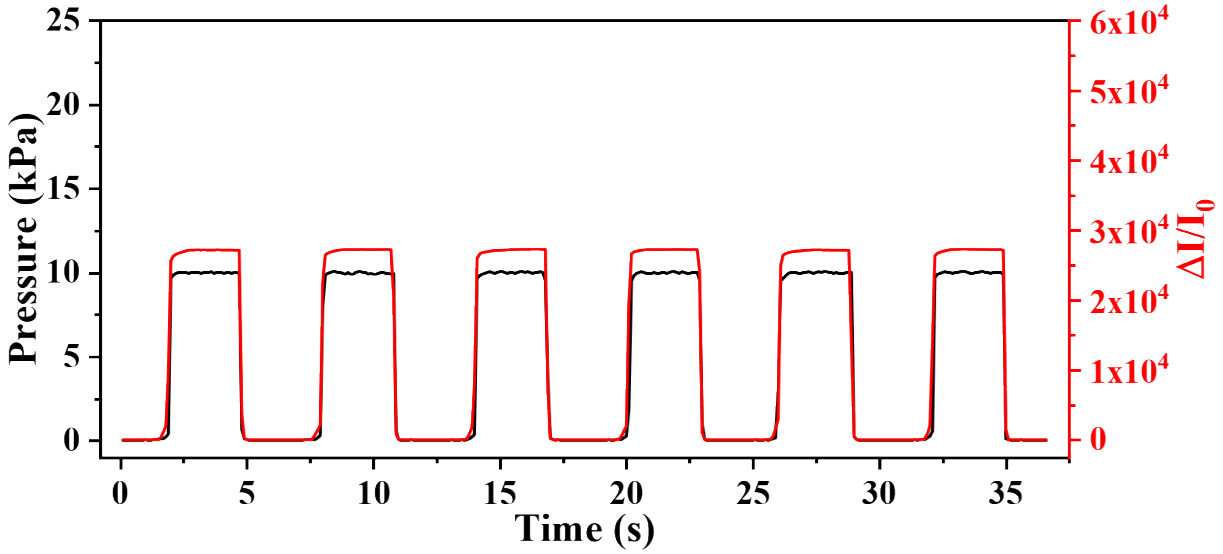

Figure S32. The real-time output sensing responses and the input pressure waves with the time evolution.

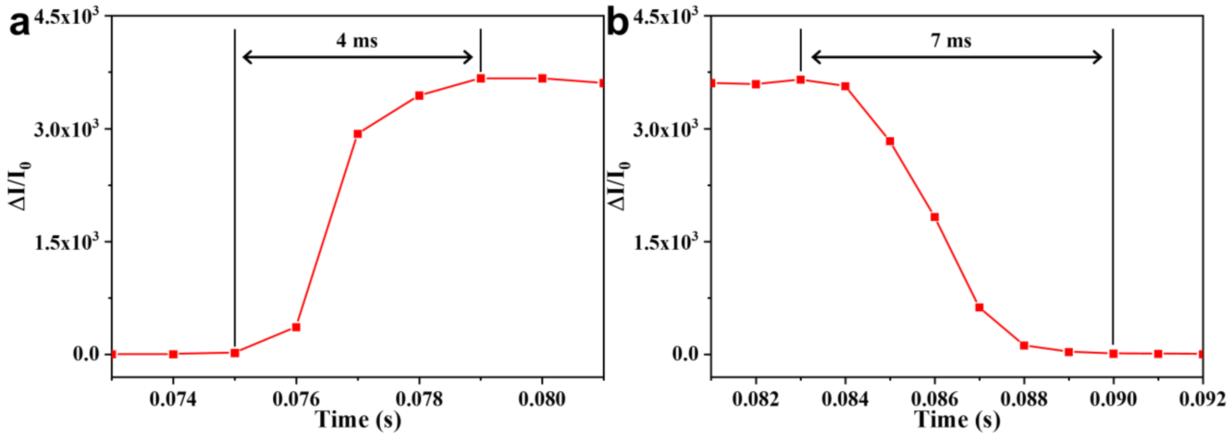

Figure S33. (a) The response time and (b) the recovery time of the flexible electronic sensor.

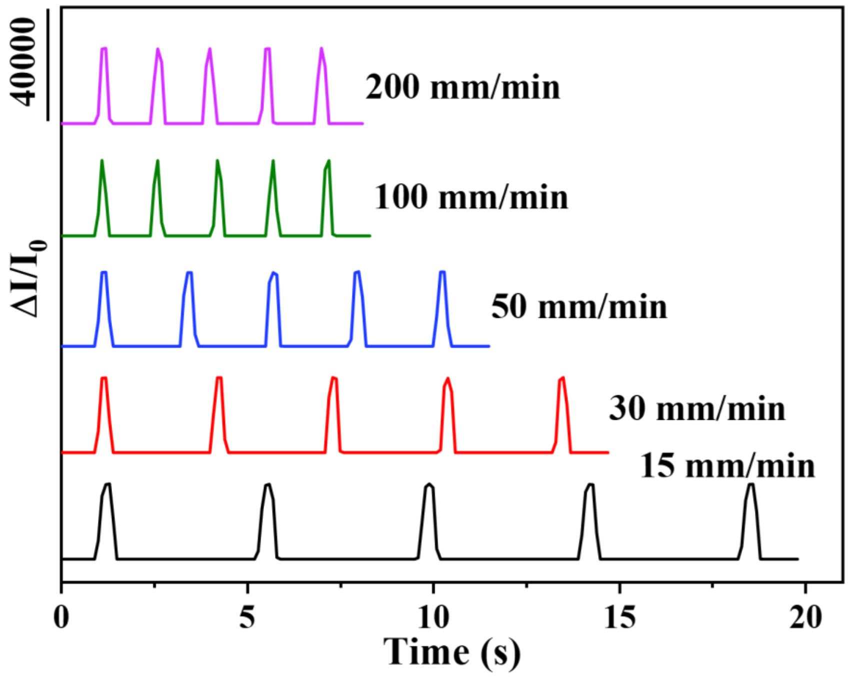

Figure S34. The sensing responses of the flexible electronic sensor under the cyclic pressure loading/unloading at different frequencies.

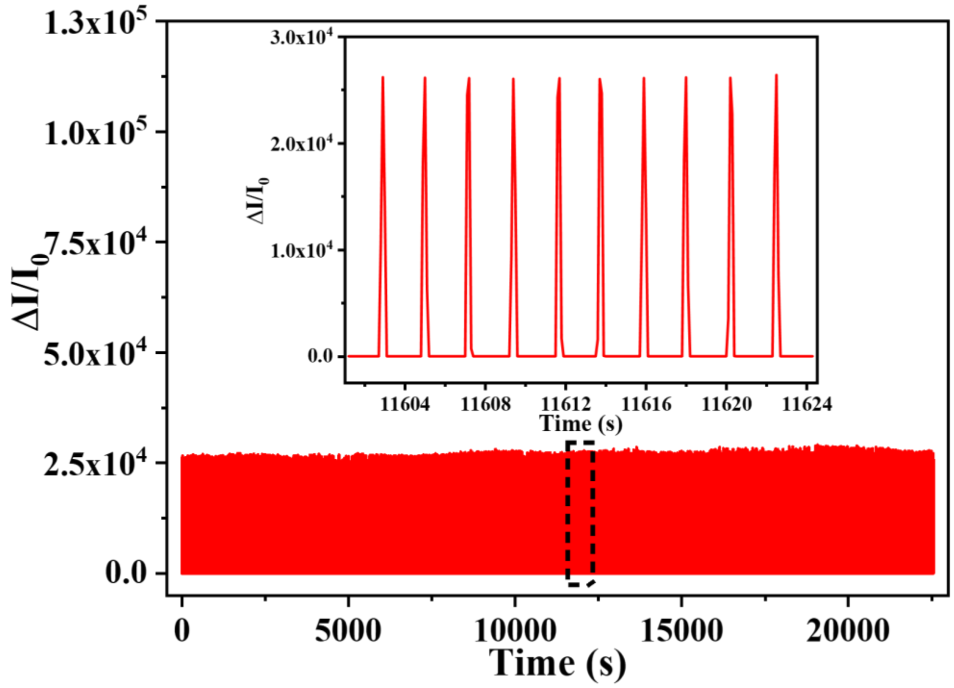

Figure S35. The cycling durability test (over 10000 cycles) of the flexible electronic sensor under the external pressure of 10 kPa.

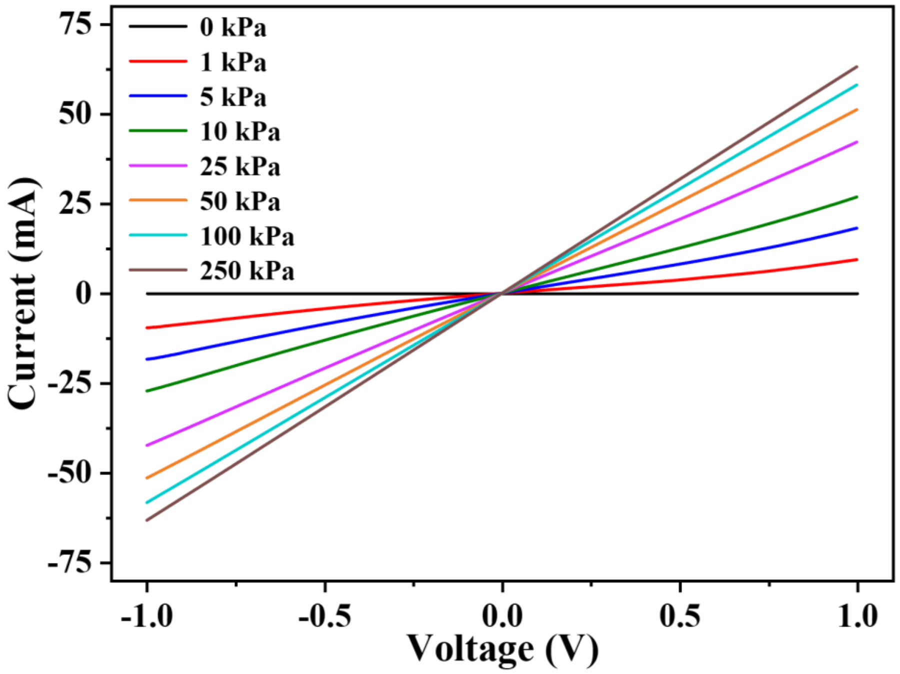

Figure S36. The I-V curves of the flexible electronic sensor at different external pressures.

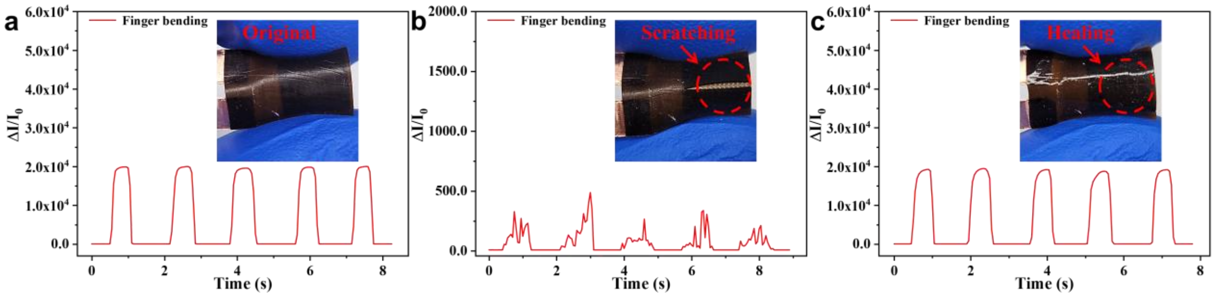

Figure S37. The sensing performances of (a) the original sensor, (b) the sensor after scratching the interdigitated electrode-coated PUPDU-Cu elastomer film of the sensor with a blade, and (c) the sensor after healing to finger bending.

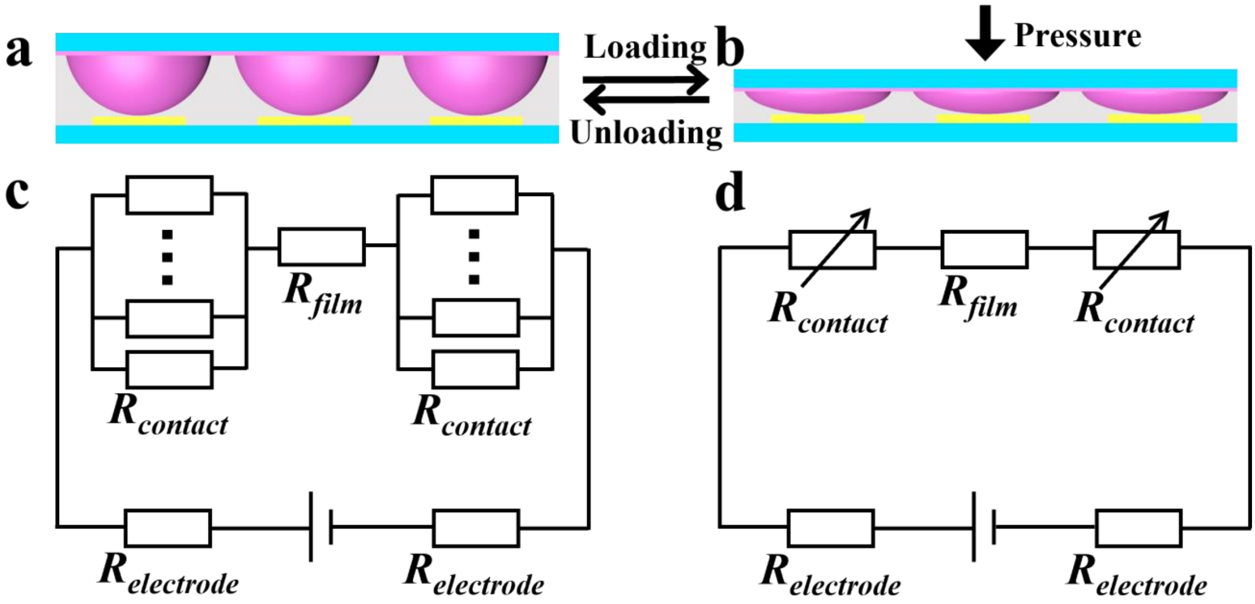

Figure S38. The sensing mechanism of the sensor (a) without and (b) with external pressure, leading to tunable changes in the contact resistance between the microdome array structure and the interdigitated electrode. The circuit schematic of the pressure sensor (c) without and (d) with external pressure.

At a fixed voltage, each contact point between the microdome array microstructure and the interdigitated electrode is equivalent to a resistor in the parallel circuit. The total resistance of the flexible electronic sensor mainly consists of three components: the resistance of the bottom silver paste pattern by screen-printing ($R_{electrode}$), the contact resistance between the microdome array microstructure and the interdigitated electrode ($R_{contact}$), and the resistance (R_{film}) of the top conductive MXene nanosheets-coated microdome array microstructure sensing film. The resistance of the silver paste is extremely smaller than that for the contact resistance between the microdome array microstructure and the interdigitated electrode ($R_{contact,}$ at the level of MΩ; $\mathrm{R}_{contact}$ >> $\mathrm{R}_{electrode}$). Thus, the $\mathrm{R}_{electrode}$ could be neglected and the change of $\mathrm{R}_{electrode}$ could be negligible from the highly slight deformation under external pressure loading. The resistance of the top conductive MXene nanosheets-coated microdome array microstructure sensing film (R_{film}, normally at several tens of Ω/□), is extremely smaller than that for the contact resistance between the microdome array microstructure and the interdigitated electrode ($R_{contact,}$ at the level of MΩ; $\mathrm{R}_{contact}$ >> R_{film}). Thus, the change of R_{film} could be negligible.

$$\boldsymbol{R_{total} = R_{electrode} + R_{contact} + R_{film} \approx R_{contact}} \quad (1)$$

The relative current change under external loaded pressure at a fixed voltage U can be expressed as:

$$\boldsymbol{\frac{\Delta I}{I_0} = \frac{I_t - I_0}{I_0} = \frac{\frac{U}{R_{total,t}} - \frac{U}{R_{total,0}}}{\frac{U}{R_{total,0}}} = \frac{R_{total,0}}{R_{total,t}} - 1 \approx \frac{R_{contact,0}}{R_{contact,t}} - 1} \quad (2)$$

$R_{total,0}$: the initial total resistance, corresponding to the initial total current I_0 of the sensor without external pressure; $R_{total,t}$: the real-time total resistance, corresponding to the real-time total current I_t of the sensor with external pressure loading; $R_{contact,0}$: the initial contact resistance of the sensor without external pressure loading; $R_{contact,t}$: the real-time contact resistance of the sensor with external pressure loading.

As reported, the resistance of the flexible pressure sensor could be expressed as $\boldsymbol{R_i = \frac{\rho L}{A_i}}$, where ρ is the resistivity of the material, L is the length (thickness) of the material, and A is the contact area between the top conductive MXene nanosheets-coated microdome array microstructure sensing film and the bottom interdigitated electrode-coated PUPDU-Cu elastomer film. Since the resistivity (ρ) and length (L) of the material are almost unchangeable, Equation (2) can be expressed as:

$$\boldsymbol{\frac{\Delta I}{I_0} = \frac{R_{total,0}}{R_{total,t}} - 1 \approx \frac{R_{contact,0}}{R_{contact,t}} - 1 = \frac{\frac{\rho L}{A_{contact,0}}}{\frac{\rho L}{A_{contact,t}}} - 1 = \frac{A_{contact,t}}{A_{contact,0}} - 1 = \frac{A_{contact,t} - A_{contact,0}}{A_{contact,0}} = \frac{\Delta A}{A_0}} \quad (3)$$

$A_{contact,0}$: the initial contact area between the top conductive MXene nanosheets-coated microdome array microstructure sensing film and the bottom interdigitated electrode-coated PUPDU-Cu elastomer film without external pressure laoding; $A_{contact,t}$: the real-time contact area between the top conductive MXene nanosheets-coated microdome array microstructure sensing film and the bottom interdigitated electrode-coated PUPDU-Cu elastomer film with external pressure loading.

Finally, the relationship between the relative contact area change at the contact interface and the sensor sensitivity can be expressed as:[1]

$$\boldsymbol{S} = \frac{\boldsymbol{\delta}\left(\frac{\boldsymbol{\Delta I}}{\boldsymbol{I_0}}\right)}{\boldsymbol{\delta P}} = \frac{\boldsymbol{\delta}[(\boldsymbol{R_{total,0}}/\boldsymbol{R_{total,t}}) - \mathbf{1}]}{\boldsymbol{\delta P}} \approx \frac{\boldsymbol{\delta}[(\boldsymbol{R_{contact,0}}/\boldsymbol{R_{contact,t}}) - \mathbf{1}]}{\boldsymbol{\delta P}} = \frac{\boldsymbol{\delta}\left(\frac{\boldsymbol{\Delta A}}{\boldsymbol{A_0}}\right)}{\boldsymbol{\delta P}} \tag{4}$$

It can be found that, under the same external pressure P loading, the sensor sensitivity is proportional to the relative contact area change between the top conductive MXene nanosheets-coated microdome array microstructure sensing film and the bottom interdigitated electrode-coated PUPDU-Cu elastomer film.

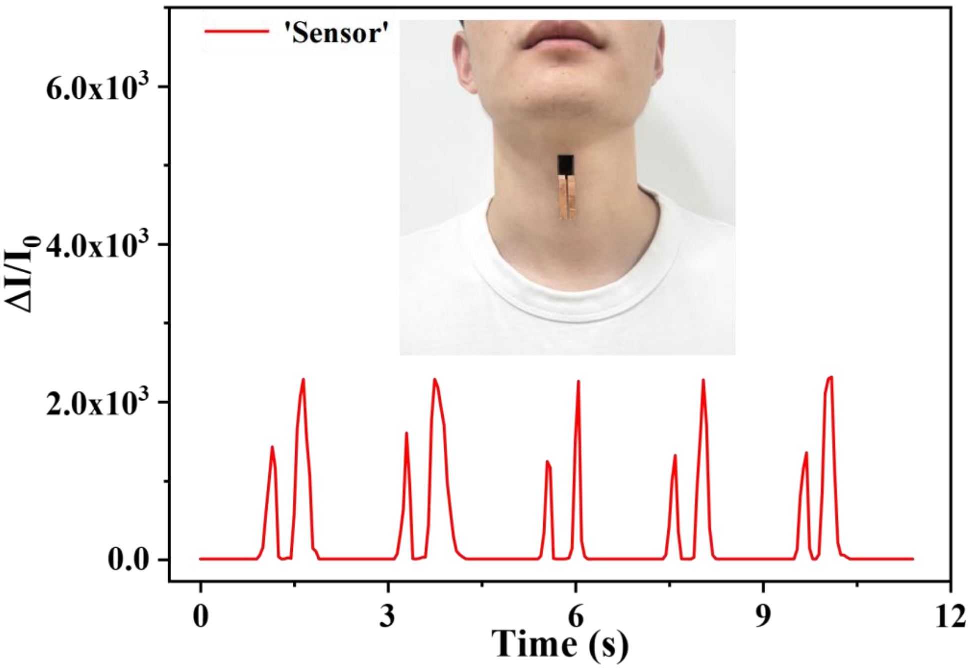

Figure S39. Sensing performance of the flexible electronic sensor when speaking "sensor" from the volunteer.

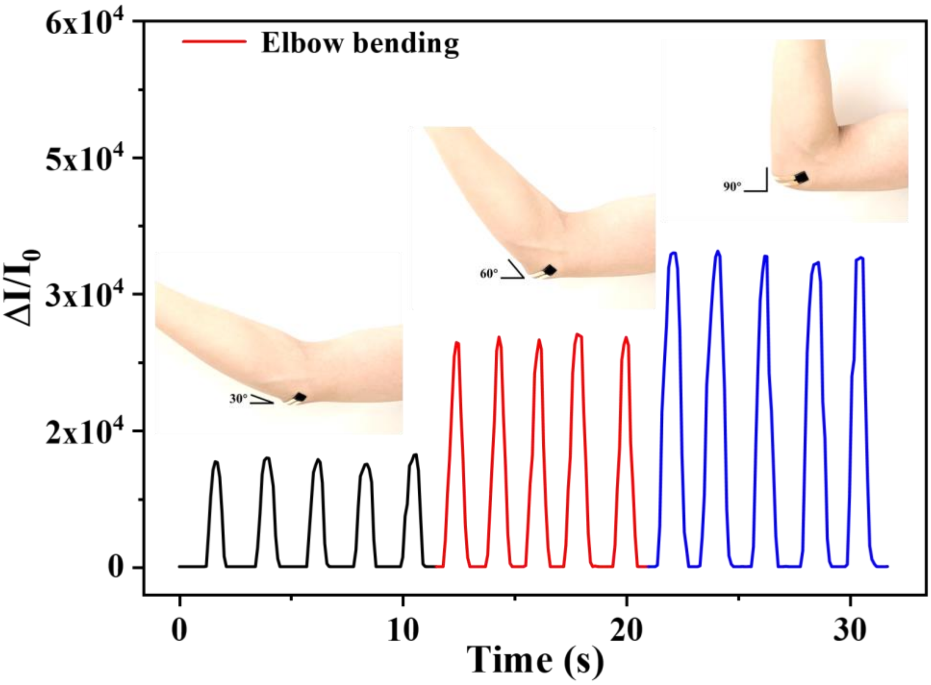

Figure S40. The sensing performance of the flexible electronic sensor to elbow bending at different angles. Inset: optical images of the flexible electronic sensor attached onto the elbow at different bending angles.

Table S1. A performance comparison of the assembled skin bionic flexible pressure sensor with other reported pressure sensors.

Sensing Materials	Maximum working range	Sensitivity	Detection limit	Response time	Cycling stability	Healability	Antibacterial	Reference
MXene/PEO	0.2 kPa	777 (0-0.02 kPa)<br>167 (0.02-0.2 kPa)	0.05 Pa	~100 ms	1000	N/A	N/A	2
Cu-FeO_x/PEN	330 kPa	18.5 (0-72 kPa)<br>38.82 (72-203 kPa)<br>154.85 (203-230 kPa)<br>34.62 (230-330 kPa)	~20 Pa	65 ms	1200	N/A	N/A	3
PU/MXene	20 kPa	281.5 (0.3-2.0 kPa)<br>509.8 (2.0-5.7 kPa)<br>66.7 (5.7-20.7 kPa)	7.8 Pa	67.3 ms	10000	YES	N/A	4
PU/IL	120 kPa	52.4 (0-1 kPa)<br>15.6 (1-10 kPa)<br>2.9 (10-100 kPa)	46 mg	32 ms	1000	YES	N/A	5
rGO/PEDOT:PSS	30 kPa	251.9 (0-100 Pa)<br>137.7 (0.1-30 kPa)	1.1 Pa	80 ms	10000	N/A	N/A	6
TPU/rGO-AgNPs/PU	40 kPa	6.57 (0-10 kPa)<br>15.91 (10-30 kPa)<br>152.97 (30-40 kPa)	~1 Pa	50 ms	10000	N/A	N/A	7
Melamine/Fe_2O_3/C@SnO_2	150 kPa	680 (0-10 kPa)<br>98 (10-50 kPa)<br>35 (50-150 kPa)	0.52 Pa	10 ms	3500	N/A	N/A	8

Table S1. Continued.

Sensing Materials	Maximum working range	Sensitivity	Detection limit	Response time	Cycling stability	Healability	Antibacterial	Reference
PDMS/PVDF/AgNWs	250 kPa	71.52 (0-75 kPa)<br>0.87 (75-250 kPa)	60 mg	25 ms	23000	N/A	YES	9
BC/PANI/CH	5 kPa	1.41 (0-0.3 kPa)<br>0.31 (0.3-1.8 kPa)	32 Pa	N/A	~80	N/A	N/A	10
Ag/PDMS	200 kPa	1.005 (0-1 kPa)<br>0.625 (1-100 kPa)<br>0.082 (100-200 kPa)	0.5 Pa	40 ms	6000	N/A	N/A	11
PU/IL/AgNWs/TiO_2	120 kPa	1.58 (0-40 kPa)<br>6.21 (40-120 kPa)	23 Pa	170 ms	6000	N/A	YES	12
PI/Paper/Borophene/IDEs	120 kPa	2.16 (0-1.2 kPa)<br>0.13 (1.2-25 kPa)<br>0.07 (25-120 kPa)	10 Pa	90 ms	1000	N/A	N/A	13
Protein/MXene	39.3 kPa	39.3 (0.089-1.071 kPa)<br>298.4 (1.4-15.7 kPa)<br>171.9 (15.7-39.3 kPa)	7.1 Pa	7 ms	10000	N/A	N/A	14
PUPDU-Cu/MXene	325 kPa	1573.05 (0.1-25 kPa)<br>160.96 (25-75 kPa)<br>21.28 (75-325 kPa)	0.98 Pa	4 ms	10000	YES	YES	This work

* PEO: poly(ethylene oxide); PEN: polyethylene naphthalate; PU: polyurethane; IL: ionic liquid; rGO: reduced graphene oxide; PEDOT:PSS: poly(3,4-ethylene dioxythiophene):poly(styrene sulfonate); TPU: thermoplastic polyurethane; PDMS: poly(dimethylsiloxane); PVDF: polyvinylidene fluoride; BC: bacterial cellulose; PANI: polyaniline; CH: chitosan; AgNWs: silver nanowires; PI: polyimide; IDEs: Au/Cr Interdigital electrodes.

References

[1] M. Lu, C. Huang, Z. Xu, Y. Yuan, M. Wang, M. Xiao, L. Zhang, P. Wan, *Adv. Funct. Mater.* **2023**, *33*, 2306591.

[2] J. Wu, X. Fan, X. Liu, X. Ji, X. Shi, W. Wu, Z. Yue, J. Liang, *Nano Lett.* **2022**, *22*, 4459.

[3] B. Feng, G. Zou, W. Wang, M. Dong, Y. Xiao, H. Ren, X. Zhao, G. Zhao, A. Wu, H. Zhu, L. Liu, *Nano Energy* **2020**, *74*, 104847.

[4] M. Yang, Y. Cheng, Y. Yue, Y. Chen, H. Gao, L. Li, B. Cai, W. Liu, Z. Wang, H. Guo, N. Liu, Y. Gao, *Adv. Sci.* **2022**, *9*, 2200507.

[5] C. Chen, W. B. Ying, J. Li, Z. Kong, F. Li, H. Hu, Y. Tian, D. H. Kim, R. Zhang, J. Zhu, *Adv. Funct. Mater.* **2022**, *32*, 2106341.

[6] H. Kong, Z. Song, W. Li, Y. Bao, D. Qu, Y. Ma, Z. Liu, W. Wang, Z. Wang, D. Han, L. Niu, *ACS Nano* **2021**, *15*, 16218.

[7] M. Cao, M. Leng, W. Pan, Y. Wang, S. Tan, Y. Jiao, S. Yu, S. Fan, T. Xu, T. Liu, L. Li, J. Su, *Nano Energy* **2023**, *112*, 108492.

[8] X. Wang, L. Tao, M. Yuan, Z. Wang, J. Yu, D. Xie, F. Luo, X. Chen, C. Wong, *Nat. Commun.* **2021**, *12*, 1776.

[9] M. Zhu, Y. Wang, M. Lou, J. Yu, Z. Li, B. Ding, *Nano Energy* **2021**, *81*,105669.

[10] J. Huang, D. Li, M. Zhao, H. Ke, A. Mensah, P. Lv, X. Tian, Q. Wei, *Chem. Eng. J.* **2019**, *373*, 1357.

[11] X. Guo, D. Zhou, W. Hong, D. Wang, T. Liu, D. Wang, L. Liu, S. Yu, Y. Song, S. Bai, Y. Li, Q. Hong, Y. Zhao, L. Xiang, Z. Mai, G. Xing, Small **2022**, *18*, 2203044.

[12] X. Cui, J. Chen, W. Wu, Y. Liu, H. Li, Z. Xu, Y. Zhu, *Nano Energy* **2022**, *95*, 107022.

[13] C. Hou, G. Tai, Y. Liu, R. Liu, X. Liang, Z. Wu, Z. Wu, *Nano Energy* **2022**, *97*, 107189.

[14] M. Chao, L. He, M. Gong, N. Li, X. Li, L. Peng, F. Shi, L. Zhang, P. Wan, *ACS Nano* **2021**, *15*, 9746.
